# A machine learning approach to assess the climate change impacts on single and dual-axis tracking photovoltaic systems

**DOI:** 10.1038/s41598-025-10831-3

**Published:** 2025-07-10

**Authors:** Udit Mamodiya, Indra Kishor, Priyam Ganguly, Isha Mukherjee, Nithesh Naik

**Affiliations:** 1 Faculty of Engineering and Technology, Poornima University, Jaipur, 303905 Rajasthan India; 2 Department of Computer Engineering, Poornima Institute of Engineering and Technology, Jaipur, 303905 Rajasthan India; 3 Energy Services, Hanwha Q Cells America Inc, Irvine, CA 92618 USA; 4 Department of Data Science, Pace University, New York, 10038-1598 USA; 5 Department of Mechanical and Industrial Engineering, Manipal Institute of Technology, Manipal Academy of Higher Education, Manipal, 576104 Karnataka India

**Keywords:** Solar tracking, Deep Q-learning, Climate adaptability, CNN-LSTM, XGBoost, Energy yield optimization, Energy infrastructure, Mechanical engineering, Energy science and technology, Engineering

## Abstract

Solar tracking system efficiency is affected by climate variability, and adaptive mechanisms must be employed to maximize energy output. Conventional fixed-tilt, single-axis, and dual-axis tracking techniques are not real-time adaptive, resulting in energy loss. This paper introduces COMLAT (Climate-Optimized Machine Learning Adaptive Tracking), an AI solar tracking system that employs climate prediction using CNN-LSTM for climate prediction, XGBoost for estimation of energy yield, and Deep Q-Learning (DQL) for real-time tracking control for solar efficiency optimization. One-year experimental research from January 2024 to January 2025 was conducted at Sitapura, Jaipur, India, with comparative studies of COMLAT and traditional tracking systems for seasonal variations and cloud cover conditions. Results confirm the 55% increase in energy production compared to fixed-tilt installations and 15–20% compared to dual-axis tracking due to its AI-based flexibility. The constructed model achieved 10-day solar irradiance forecasting with an RMSE of 23.5 W/m², outperforming the conventional LSTM and GRU baselines. XGBoost made predictions of energy output with an R² score of 0.94. COMLAT’s reinforcement learning controller optimized tracking angles with sub-second latency while minimizing mechanical movement. The integration of hybrid artificial intelligence models allows COMLAT to continuously update its tracking angles in real time and is a scalable and industrially viable solution for smart grids, solar farms, and hybrid renewable energy systems. Increasing computational efficiency, integrating energy storage mechanisms, and optimizing self-learning algorithms will be areas of focus for future research to make it more applicable.

## Introduction

The transition towards sustainable energy alternatives has accelerated research and development (R&D) in solar photovoltaic (PV) technology, driven by the need for greater efficiency, cost, and climatic flexibility. Solar energy plays an increasingly prominent role in the global energy landscape; however, its inherent intermittence and vulnerability to geographical and weather conditions hamper the achievement of maximum energy conversion efficiency^1–3^. A PV system depends heavily on how much sun it receives, and this varies seasonally, by atmospheric conditions, and daily sun movement. Fixed-tilt solar panels most often stand stationary at their optimal tilt but lose efficiency through non-optimal exposure to the sun. To overcome these disadvantages, solar tracking systems have been developed, in which PV panels follow the sun’s path to maximize the utilization of power generation throughout the day^5,6^. Typical solar tracking devices are single-axis and dual-axis tracking systems. Single-axis trackers pivot the azimuth angle to follow the east-west motion of the sun, producing 25–30% more energy than fixed-tilt panels. Dual-axis trackers, which tilt and move both azimuth and angles, improve efficiency by a further 40–45% but are linked with greater mechanical complexity, maintenance, and greater energy consumption for motorized motion. The innovation of COMLAT is that it can dynamically change between static, single-axis, and dual-axis tracking and real-time adjustment as a function of the forecast and actual weather. The CNN-LSTM forecasts solar irradiance, temperature, and cloud motion and generates an optimized tracking prediction system. The XGBoost model calculates the energy yield that will be projected as a function of different tracking and then determines the amount of trade-off to increase the energy gain against the mechanical motion cost^7–10^. Finally, Deep Q-Learning (DQL) learns to select the most optimal tracking mode autonomously, ensuring that the system extracts the highest quantity of energy using the lowest computational and mechanical overhead. This hybrid AI solution allows COMLAT to operate more efficiently, with maximum adaptability and minimum energy loss under extreme weather conditions and therefore qualifies as a next-generation solution for smart solar tracking. This study develops and experimentally validates COMLAT as an AI-based adaptive solar tracking system that integrates machine learning and reinforcement learning techniques to optimize solar panel orientation in real time. The overall objective was to develop a climate-resilient tracker system that responds adaptively to changing environmental conditions, solar radiation patterns, and variations in cloud cover^11^. An experimental one-year study (January 2024 – January 2025) was conducted in Sitapura, Jaipur, India, where COMLAT performance was compared with traditional fixed-tilt, single-axis, and dual-axis tracker systems to examine energy yield gains, tracking accuracy, and computational expense^12–15^. The primary research interest of this paper was to compare COMLAT’s AI-driven tracking optimization with traditional mechanical tracking, emphasizing its excellence in real-time responsiveness, computational speed, and predictive decision-making. Research compared the computational overhead of AI-driven tracking and explored how to minimize the processing delay while optimizing tracking efficiency. In addition, the research examines application of COMLAT in optimizing overall energy production in various seasonality and reducing loss of energy in cloudy or low-radiation days. To address these issues, COMLAT is introduced here as an industrial-scale and scalable technology for renewable energy systems with applications in smart grids, large-scale solar farms, and energy networks. This study offers several new contributions to AI-based solar tracking systems with significant improvements in energy efficiency, tracking adaptability, and computational intelligence. This study introduces a hybrid AI-based solar tracking system that integrates CNN-LSTM, XGBoost, and deep Q-learning (DQL) for predictive and real-time tracking adjustments. Compared to conventional tracking systems that trace fixed or semi-fixed movement paths, COMLAT increasingly chooses the most energy-efficient tracking method dynamically, maximizing the overall solar power production by up to 55% compared to fixed-tilt systems. Another major contribution is the development of a climate-aware AI model with CNN-LSTM for real-time weather forecasting to forecast variations in solar irradiance, temperature, and cloud cover. Based on this predictive model, COMLAT can make proactive adjustments, thereby significantly reducing energy losses during unpredictable weather conditions^12–17^. Moreover, this study provides an experimental validation of COMLAT under real conditions over one year of observation, pitting its performance against seasonal variation, extreme temperature variations, and cloud cover. The system is also contrasted with existing fixed, single-axis, and dual-axis tracking systems and shows that it is more efficient, flexible, and resilient than the existing systems. Second, the research also adds to AI-optimization of energy through the application of the Deep Q-Learning (DQL) algorithm to real-time tracking control, which is not normally part of existing solar tracking models. The reinforcement learning process enhances the optimal tracking processes with time and allows COMLAT to optimize its decision-making process step by step without any external feedback. The adaptation and optimization capabilities of the system allow it to be highly scalable for smart solar grids, autonomous renewable power management, and future AI-based energy systems. With its proof of feasibility, computability, and energy efficiency, this work makes COMLAT a next-generation AI-driven solar tracking technology bridging the gap between mechanical trackers of yesteryears and smart, autonomous energy optimizing technology.

The novelty of the work is the development and implementation of COMLAT (Climate-Optimized Machine Learning Adaptive Tracking), a shared artificial intelligence platform that processes in parallel climate forecasting, energy generation forecast, and intelligent real-time tracking control. Unlike existing research on individual aspects such as irradiance estimation or mechanical tracking, COMLAT integrates CNN-LSTM for climatic prediction, XGBoost for power prediction, and Deep Q-Learning (DQL) for intelligent tracking mode control under a single self-tuning architecture. The system adapts dynamically to tilt and azimuth angles based on climate change, outperforming traditional static and two-axis trackers. Moreover, the system is thoroughly experimented with a 1-year long experimental real-world dataset to ensure its practicability. Its real-time behavior (< 1 s), season agility, and low computational costs by employing lightweight edge AI models make COMLAT an innovative and scalable solution for future climate-resilient solar farms.

## Literature review

Advances in solar tracking technologies have enhanced photovoltaic (PV) generation as solar panel efficiency relies significantly on the incidence angle of solar radiation. The use of fixed-tilt, single-axis, and double-axis trackers has, in the past, been utilized in preference to fixed deployments^1^. The conventional trackers don’t real-time adjust, utilize forecasted cloud and wind patterns, or operate to maximize alternative seasons^2^. Advances in machine learning (ML) and artificial intelligence (AI) have made it such that the upcoming systems are in a position to self-tune in tracking modes, solar irradiance prediction, and optimization of real-time energy harvesting based on environmental conditions^3^. This literature review addresses the limitations of conventional solar tracking systems, the advent of AI-driven tracking technology, advances in solar energy harvesting automation predictive modeling, and the use of reinforcement learning to enable smart control automation. In addition to the challenges presented by AI-driven systems when compared to non-tech options, there is also a significant focus on creating AI-driven solar tracking systems for large installations. Fixed-tilt photovoltaic installations are the largest installed type of PV installations. It exhibits low performance levels owing to its fixed design^4^. The system is plagued with high energy loss due to inefficient exposure to sunlight in various parts of the day and year. To overcome such inefficiencies, single-axis tracking systems have been introduced, where panels can align themselves along a single degree of freedom (commonly east-west)^5^. These systems can boost energy generation by up to 30% over fixed-tilt panels but still fall short in the event of seasonal variations or sudden weather patterns. Dual axis tracking systems, which change both tilt and azimuth angles, provide a better solution by providing maximum solar irradiance capture during the day and season^6^. Higher mechanical complexity, operational costs, and motorized energy consumption reduce their long-term viability, especially in large solar farms^7^. The most significant disadvantage of traditional tracking systems is that they rely on preprogrammed movement schemes^8^. This is not accountable for real-time variability in cloud cover, humidity fluctuations, and changes in direct normal irradiance (DNI). Traditional tracking systems do not dynamically adjust to cloud cover, humidity fluctuations, or DNI variability^9^. Furthermore, unwanted mechanical motion can cause wear and tear, increased maintenance, and system downtime. The need for a smarter, self-learning solar tracking system has spurred the evolution of AI-based solar tracking systems to maximize panel alignment from real-time environmental monitoring and forecasting models^10,11^. Use of AI in solar tracking systems has revolutionized efficiency optimization using data-driven decision making, predictive analysis, and real-time adjustment. Machine learning algorithms, deep learning, and reinforcement learning have been employed in AI-driven solar trackers to make automatic panel angle adjustments according to weather forecasts as inputs, past trends, and sensor inputs^12^. These systems avoid real-time realignment and predictive control of trackers because they adjust real-time. Deep learning techniques such as Convolutional Neural Networks (CNNs) are used extensively in the real-time detection of cloud cover to allow solar trackers to forecast the variation in irradiance and move the panels accordingly^13,14^. Long Short-Term Memory (LSTM) networks, which are a specific type of recurrent neural network (RNNs), are well fitted to time-series forecasting in solar irradiance forecasting, such that solar tracking systems can make movement adjustments based on projected future energy yield in advance rather than reactive adjustments. Machine learning regression models, including XGBoost and Random Forest algorithms, are used to accurately estimate projected power generation under different meteorological conditions, enabling decision-making by tracking systems for energy-efficient positioning^15^. Reinforcement learning-based methods also improve solar tracking efficiency by facilitating autonomous decision-making with respect to changing environmental conditions. Deep Q-learning (DQL), a reinforcement learning technique, enables solar trackers to progressively improve their tracking strategy such that panels are always run at optimal efficiency with the least amount of unnecessary movement and mechanical wear^16^. –^17^ The capacity of AI-based systems to integrate several predictive models, respond to real-time sensor data, and learn tracking strategies on their own makes them the most viable method for optimizing solar tracking. These systems keep solar panels under the best possible working conditions at all times, resulting in dramatic enhancements in energy output and system life^18,19^. Solar tracking capability is extremely dependent on environmental parameters because solar irradiance varies in accordance with cloud cover, atmospheric humidity, pollution, and yearly changes^20 ,21^. Typical tracking systems fail to adapt because of these environmental parameters, thus providing less than optimal energy levels under low radiation levels^22,23^. AI-controlled tracking technologies feature climate-adaptive properties, thereby maintaining the panels’ position at maximum levels even under fast-changing patterns of weather conditions^24,25^. To accomplish this, hybrid AI models integrating CNN-based cloud detection, LSTM-based irradiance forecasting, and reinforcement-learning-based tracking control have been implemented in contemporary solar tracking systems^26,27^. Hybrid systems ensure that solar panels make proactive adjustments based on expected climate conditions instead of responding after inefficiencies are experienced. These methods drastically minimize the power losses resulting from dynamic environmental variations and increase the overall reliability of solar energy production^28,29^. The use of AI-powered solar tracking leads to computational issues, particularly in handling real-time decision-making with efficiency^30,31^. Deep and reinforcement learning models require high processing capabilities, which could be a major bottleneck in large-scale implementations^32,33^. The issue is balancing the computational efficiency and optimization of tracking so that tracking decisions are carried out with little delay and energy consumption^34^. To address this, edge computing frameworks have been implemented in AI-driven solar trackers to enable real-time processing at the sensor level instead of using cloud-based AI models^35,36^. This results in a much lower latency and enhances real-time adaptability, making AI-driven tracking viable for large solar farms^37^. Furthermore, model compression methods and hardware-aware AI optimizations are being considered to reduce computational overhead while maintaining predictive accuracy^38^. The ongoing development of AI for solar tracking will be augmented by self-learning models, federated AI architectures, and quantum computing implementations, further improving the tracking precision and efficiency^39^. Future solar trackers will utilize multimodal AI, combining satellite-based weather model data, Internet of Things (IoT) sensors, and energy demand forecasting models^40^. AI will also be instrumental in hybrid renewable energy systems, managing the optimal power distribution among solar, wind, and battery storage systems^41^. Next-generation systems, as AI-based tracking continues to advance, will be completely autonomous, requiring little human input to ensure that solar energy harvesting is at maximum possible efficiency under all conditions^42^. AI-based solar tracking is a paradigm change in renewable energy optimization that transcends preprogrammed mechanical tracking mechanisms to self-adaptive, smart solar optimization models. Deep learning, reinforcement learning, and hybrid AI models have been found to be superior in terms of energy efficiency, flexibility, and computational smartness, making AI-based solar tracking the next innovation in green-energy technology.

### Literature gap

An in-depth survey of the current solar tracking literature reveals key shortcomings that limit the maximum realization of adaptive high-efficiency solar tracking systems. Conventional tracking systems use preprogrammed motion patterns, are not adaptive to environmental conditions, and are unable to dynamically optimize tracking tactics. AI-driven tracking has also been an evolving prospect, but there are a few prominent challenges yet to be met, such as climate adaptability, intelligence in decision making, computational efficiency, optimization of energy yields, and real-world testing. This study introduces Climate-Optimized Machine Learning Adaptive Tracking (COMLAT) as an innovative AI-driven solar tracking system that resolves the aforementioned shortcomings systematically through prediction analytics, reinforcement learning-based decision-making, and real-time optimization methods.

#### Lack of climate-adaptive tracking for dynamic weather conditions

Current solar tracking systems follow fixed or semi-fixed movement patterns and do not dynamically adapt to actual climatic changes such as cloud cover, humidity, and seasonal changes. Traditional models do not use predictive analytics to estimate solar irradiance, resulting in energy losses during sudden weather changes.

#### Lack of AI-based decision-making for best tracking mode selection

The majority of traditional tracking systems operate in one predefined mode, such as static, single-axis, or dual-axis tracking, with no capacity to dynamically change between modes depending on the current conditions. Although certain AI-based tracking models optimize tilt angles, they do not utilize reinforcement learning for automatic mode selection, which reduces their adaptability.

#### High computational overhead in AI-based solar tracking systems

AI-based solar tracking models involve computational complexity with the need for high-performance computation units (GPUs/CPUs) to execute deep learning algorithms. Classic AI-based systems are computationally intensive, thus making real-time solar tracking computationally costly and difficult to deploy in mass market applications.

#### Restricted integration of solar tracking with energy yield prediction models

The majority of tracker systems do not incorporate energy production estimation into optimization models but instead employ solar position-dependent tracking systems. This generates scenarios in which the solar energy yield benefits are cancelled by unnecessary mechanical action and energy drain.

#### Failure of experimental verification of AI-based solar tracking systems

Most solar tracking research using AI depends on simulated data with no field deployment; thus, it is challenging to test the long-term reliability, scalability, and real-time efficiency. The lack of field experiments makes the practicality of AI-based tracking systems in real-world usage uncertain.

## Methodology

### Proposed work

The proposed research concentrates on creating an adaptive solar tracking system powered by AI that adjusts the orientation of solar panels according to the current climate conditions and seasonal changes over a period. The system evaluates the effect of climate change on the efficiency of solar tracking for static, single-axis, and dual-axis tracking systems using a 330 W solar panel setup. The system, by combining machine learning-based prediction and reinforcement learning-based optimization, dynamically selects the most efficient tracking mode to maximize energy output while reducing energy consumption.

The main goals of this study are:

Formulating an adaptive tracking algorithm that dynamically adjusts panel orientation based on real-time weather patterns and climate trends. To Evaluating the effects of seasonal climate changes, such as temperature, solar irradiance, cloud cover, wind speed, and humidity, on the performance of static, single-axis, and dual-axis tracking systems for a period of one year (January 2024–January 2025). To Apply a hybrid machine learning strategy that combines the convolutional neural network–long short-term memory (CNN–LSTM) for solar irradiance and climate forecasting. The XGBoost Regression for the estimation of energy yield based on various tracking modes.Deep Q-learning (DQL) was used to optimize real-time tracking modes based on energy efficiency forecasting and climate conditions. Tuning the performance of the ML-based adaptive tracking system compared with traditional tracking schemes to assess energy efficiency improvement and system resilience against climate change.

**Fig. 1:**
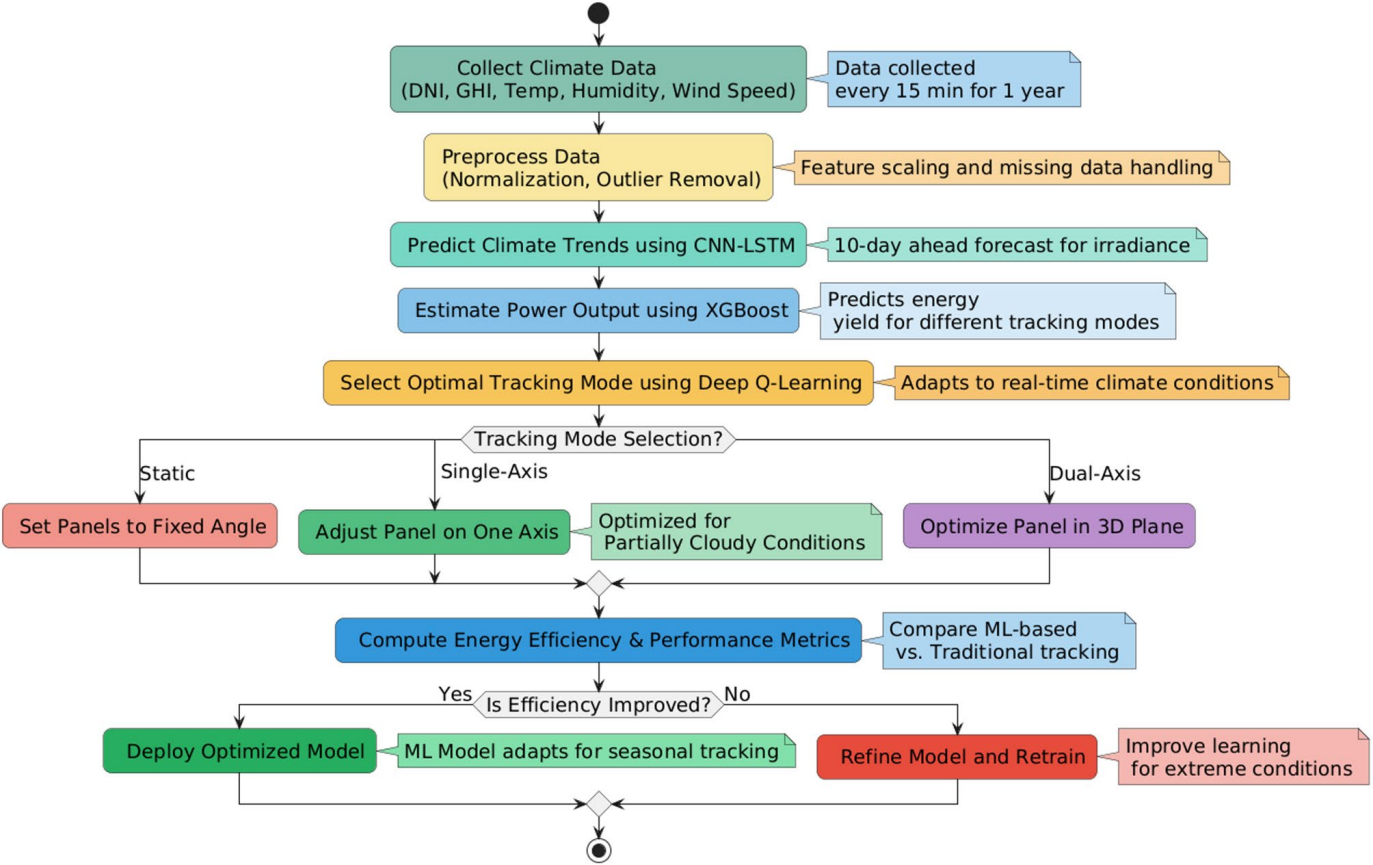
Flowchart of methodology for AI-based adaptive solar tracking system. The process integrates climate data collection, machine learning-based climate prediction, estimation of energy yield, and reinforcement learning-based tracking optimization to enhance the efficiency of solar energy.

This study makes a novel contribution that is distinct from the classic solar tracking solutions. Climate predictions and adaptive reinforcement learning enable instant solar tracking maximization. Compared with classic trackers based on programmed algorithms, this system has an automatic adaptation capability that follows season-changing and instant-on weather updates. The use of the reinforcement model enables learning through self-practice and maximization, without requiring regular manual re-adaptation for optimal results and decision-making. Figure 1 depicts the well-defined workflow of the proposed AI-driven adaptive solar tracking system. The approach was initiated with the real-time acquisition of climate parameters, such as solar irradiance, temperature, humidity, wind speed, and cloud cover. Preprocessing of the collected data ensures normalization, elimination of outliers, and feature extraction to support accurate machine learning prediction. The CNN-LSTM model thereafter predicts climate patterns, which supports predictive insights regarding solar irradiance variability. Next, the Boost regression model predicts the energy yield in varying tracking modes to form a basis for decision making. The Deep Q-Learning model selects the best tracking mode (static, single-axis, or dual-axis) with the maximum efficiency in real-time climate input and forecasted power output. The system was continuously analyzed to enhance the model to fit seasonal patterns and maximize the long-term tracking efficiency. This method ensures that solar panels dynamically adjust to fluctuating weather patterns, leading to greater energy efficiency and reduced power usage, which aligns with the research objective of developing a climate-resilient solar-tracking optimization system.

The proposed AI-driven decision system integrates CNN-LSTM for climate prediction, XGBoost for power calculation, and Deep Q-Learning for tracking mode choice. This system guarantees greater adaptability and efficiency enhancement compared to traditional tracking systems. The proposed AI-powered tracking model scales with the ability to integrate it into large-scale solar farms and uses IoT-based edge computing for real-time decisions.

### Data collection & preprocessing

The data applied in this research is one year (January 2024–January 2025) of climate and energy data of Sitapura, Jaipur, India. The data were derived from meteorological data, on-site sensor measurements, and historical data to present an extensive climate observation and accurate machine learning prediction.

#### Dataset description

The dataset of research includes the following key climate and energy parameters:

- Solar irradiance data: The Direct Normal Irradiance (DNI), Global Horizontal Irradiance (GHI), and Diffuse Horizontal Irradiance (DHI), which are recorded in every 15-minute intervals that allow assessment of the solar energy potential at different times of the day.
- Temperature & humidity: Measured hourly intervals to account for seasonal fluctuations that impact solar panel efficiency.
- Wind speed & Cloud cover: The Data obtained from weather stations and satellite images that evaluate the effect of environmental factors on the tracking performance.
- Energy output: The Collection from the three different tracking systems (a) static, (b) single-axis, and (c) dual-axis tracking, which logged at 30-minute intervals to compare their performance across varying climatic conditions.

**Table 1:** Summary of collected climate and energy data utilized for AI-based adaptive solar tracking.

Parameter	Symbol	Unit	Data source	Recording frequency	Purpose in study
Direct normal irradiance	DNI	W/m²	Pyranometer, Satellite	Every 15 min	Solar energy potential analysis
Global horizontal irradiance	GHI	W/m²	Pyranometer, Weather Station	Every 15 min	Evaluating total received solar radiation
Diffuse horizontal irradiance	DHI	W/m²	Pyranometer, Weather Station	Every 15 min	Understanding scattering effects
Ambient temperature	T	°C	Temperature Sensor	Hourly	Impact on panel efficiency
Relative humidity	RH	%	Humidity Sensor	Hourly	Correlation with energy yield
Wind speed	WS	m/s	Anemometer, Weather Station	Hourly	Effect on tracking stability
Cloud cover index	CCI	%	Satellite Imagery	Hourly	Input for CNN-LSTM model
Energy output (Static)	P_static	kWh	Power Meter (Solar Panel)	Every 30 min	Performance of fixed panels
Energy output (Single-Axis)	P_single	kWh	Power Meter (Solar Panel)	Every 30 min	Efficiency of single-axis tracking
Energy output (Dual-Axis)	P_dual	kWh	Power Meter (Solar Panel)	Every 30 min	Efficiency of dual-axis tracking
Solar panel tilt angle	θ	Degrees	IMU Sensor (Tracking System)	Real-time (Dynamic)	Panel orientation optimization

The dataset includes solar irradiance, temperature, humidity, wind speed, cloud cover, and energy output for different tracking modes, recorded over a period of one year (January 2024–January 2025) in Sitapura, Jaipur, India.

**Table 2:** Typical sample of climate and energy data for different seasonal variations (Winter, summer, monsoon, and Post-Monsoon) for solar tracking using AI.

Timestamp	DNI (W/m²)	GHI (W/m²)	DHI (W/m²)	Temp (°C)	Humidity (%)	Wind Speed (m/s)	Cloud Cover (%)	P_static (kWh)	P_single (kWh)	P_dual (kWh)	Tilt Angle (°)	Season
01-01-2024 10:00	540	620	80	18	55	2.3	10	2.5	3.1	3.8	25	Winter
01-01-2024 12:00	810	900	90	22	50	3.2	5	3.8	4.5	5.2	40	Winter
01-01-2024 14:00	750	830	80	24	48	2.8	12	3.5	4.1	4.9	35	Winter
01-05-2024 10:00	900	980	90	35	30	2	2	3.5	4.6	5.3	45	Summer
01-05-2024 12:00	1050	1150	100	42	25	3.1	0	4.8	5.7	6.5	50	Summer
01-05-2024 14:00	980	1060	90	44	22	3.5	5	4.3	5.2	6.1	48	Summer
01-07-2024 10:00	300	350	150	28	80	4.5	70	1.2	1.8	2.3	20	Monsoon
01-07-2024 12:00	450	500	180	30	85	5	85	1.7	2.3	3.1	22	Monsoon
01-07-2024 14:00	400	450	170	31	87	5.2	90	1.5	2	2.7	21	Monsoon
01-10-2024 10:00	600	670	80	26	60	3	25	2.8	3.5	4.1	30	Post-Monsoon
01-10-2024 12:00	850	920	90	30	55	3.8	12	4.1	5	5.7	38	Post-Monsoon
01-10-2024 14:00	790	860	85	32	50	3.5	18	3.8	4.7	5.3	36	Post-Monsoon

The data pertain to solar radiation, climatic conditions, and power output for static, single-axis, and dual-axis trackers, to represent the impact of climate variation on solar panel efficiency. Tables 1 and 2 provide a clear presentation of the datasets used in this study. Table 1 summarizes the climate and energy parameters measured, including solar irradiance (DNI, GHI, and DHI), temperature, humidity, wind speed, cloud cover, and energy generation for different tracking modes to provide full data coverage for machine learning-based optimization and forecasting. Table 2 presents example data collected over different seasons (Winter, Summer, Monsoon, and Post-Monsoon) that reflect the variations in solar radiation, climatic conditions, and energy output of the static, single-axis, and dual-axis tracking mechanisms. This information is important for training and validating AI models to take climate-respondent tracking decisions in accordance with real-time conditions.

#### Justification for using Jaipur as the study site

Jaipur, India, is an ideal location for this research for the many reasons, the location has high solar irradiance zone, In Jaipur experiences an average DNI of ~ 5.5 kWh/m²/day, making it well suited for evaluating solar tracking efficiency. It has Distinct Seasonal Variations, in this region experiences extreme summers (~ 45 °C), cold winters (~ 5 °C), and monsoon seasons, providing diverse climate conditions to test the adaptive tracking performance. Existing Solar Installations in the Jaipur has several operational solar farms that facilitate real-world experimental validation and comparison with conventional tracking methods.

#### Data processing steps

For high-quality and consistent data, the following preprocessing methods are used:

- Handling missing values: Missing values caused by sensor faults or communication loss are treated using linear interpolation for short missing intervals and statistical modeling for long missing durations to ensure data consistency.
- Feature scaling & normalization: Min-Max normalization is used to scale all input variables to a common range (0 to 1) to avoid bias in machine learning models.
- Outlier detection & removal: Filtering based on Z-scores is employed to remove abnormal readings that can be caused by unusual weather conditions or faulty sensors.
- Data augmentation for machine learning: Synthetic data generation methods are utilized to improve model robustness to ensure that the ML model generalizes well to new climatic conditions.

#### Seasonal variability and cloud cover impact on solar tracking efficiency

**Fig. 2:**
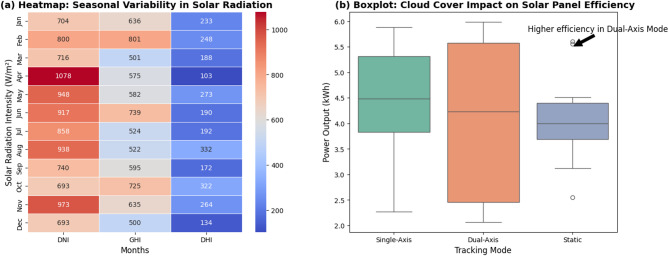
(**a**). Heatmap of seasonal solar radiation variation, illustrating monthly variations of Direct Normal Irradiance (DNI), Global Horizontal Irradiance (GHI), and Diffuse Horizontal Irradiance (DHI). (The visualization was generated using Seaborn v0.12.2 (https://seaborn.pydata.org/) and Matplotlib v3.7.1 (https://matplotlib.org/). The dataset was processed using Gaussian Kernel smoothing and Min-Max Scaling to enhance clarity.)

The seasonal variation and cloud cover effect on the solar tracking efficiency are pivotal in the optimization of the adaptive positioning of solar panels. Seasonal changes in Direct Normal Irradiance (DNI), Global Horizontal Irradiance (GHI), and Diffuse Horizontal Irradiance (DHI) influence power output; hence, predictive climate simulation was used to optimize tracking efficiency. These variations are illustrated in Fig. 2(a) through a heatmap in which solar radiation on a monthly basis is plotted and labeled as varying with months and directly influencing the energy output of the single-axis, static, and dual-axis tracker systems. The boxplot representation of cloud cover impact in solar tracking mode is complemented by Fig. 2(b) and shows that static panels lose more efficiency in a cloudier period, whereas the dual-axis tracker possesses relatively consistent power output. These findings emphasize the need for machine-learning-based tracking decisions according to season and instantaneous weather conditions to ensure enhanced energy efficiency under dynamically changing environments.

Figure 2 illustrates the impact of seasonal variations on the available solar energy level, which is important for enhancing adaptive solar tracking methods. (b). Boxplot of the impact of cloud cover on solar panel efficiency for different tracking modes (static, single axis, and dual axis). The plot shows the variation in power generation due to changing cloud cover, which helps to optimize machine learning-based optimization techniques for decision tracking. Preprocessed data form the foundation for climate forecasting, estimation of energy yield, and real-time adaptive tracking, giving the proposed system a way of effectively responding to changing climate patterns and optimizing solar panel efficiency.

### Climate impact prediction using CNN-LSTM

Solar irradiance variations and climate variability must be forecast to optimize adaptive solar tracking systems. Energy production is considerably affected by seasonal cycles, cloud cover, and temperature variations, making it crucial for an AI-based predictive system to be in place. A 10-day forecast of solar irradiance trends is provided by a hybrid CNN-LSTM model so that the solar panel orientation may be adaptively controlled in real time.

#### Model selection & justification

The proposed deep learning model integrates Convolutional Neural Networks (CNN) and Long Short-Term Memory (LSTM) to capture spatiotemporal dependencies in climate patterns.

CNN for feature extraction:

- Input: Satellite cloud cover images of size 256 × 256 × 3 (RGB bands).
- Architecture: Three convolutional layers with kernel size 3 × 3 and ReLU activation.
- Pooling Layer: Max pooling (2 × 2) to reduce spatial dimensions.
- Feature Vector Output: Flattened to 512 features for LSTM integration.

LSTM for Time-Series Forecasting:

- Input: Climate feature matrix X_t_​ with past 30 days of solar irradiance, temperature, and humidity.
- Architecture: Two LSTM layers with 128 hidden units each.
- Dropout: 20% (0.2) to prevent overfitting.
- Activation function: Tanh (for sequential dependencies).
- Output: 10-day forecast of DNI, GHI, and DHI values.

#### Mathematical model for solar irradiance prediction

The solar irradiance prediction model is mathematically expressed as in Eq. (1):

1 $$\:\text{I}\left(\text{t}\right)={\text{C}\text{N}\text{N}}_{\text{f}\text{e}\text{a}\text{t}\text{u}\text{r}\text{e}\text{s}}\text{}\left({\text{X}̅}_{\text{t}}\text{}\right)+\text{L}\text{S}\text{T}\text{M}\left({\text{W}̅}_{\text{t}}\right)$$

Where, I(t) = Predicted solar irradiance at time t̅, $\:{\text{X}̅}_{\text{t}}$ = Climate feature matrix (Temperature, Cloud Cover, Humidity) and $\:{\text{W}̅}_{\text{t}}$​ = Past 30 days of irradiance values for LSTM-based forecasting.

The loss function used for the optimization is the Mean Squared Error (MSE), as shown in Eq. (2):

2 $$\:\text{M}\text{S}\text{E}=\frac{1}{n}\:\sum\:_{i=1}^{n}{\left({\text{I}}_{\text{a}\text{c}\text{t}\text{u}\text{a}\text{l}}\text{}\right(\text{t})-{\text{I}}_{\text{p}\text{r}\text{e}\text{d}\text{i}\text{c}\text{t}\text{e}\text{d}}\text{}(\text{t}\left)\right)}^{2}\:\:\:\text{}$$

where I_actual_​ is the true irradiance value, and I_predicted​_ is the CNN-LSTM forecasted irradiance. The model was trained using the Adam optimizer with an initial learning rate of 0.001 and dynamically adjusted using a learning rate decay factor to prevent overfitting.

##### Expected output and uncertainty analysis

The new CNN-LSTM model is predicted to produce a high-precision 10-day ahead solar irradiance forecast, which can be utilized directly for energy yield estimation and adaptive tracking mode choice. However, owing to the natural unreliability of climate forecasting, confidence intervals of predictions must be evaluated to estimate potential variations from real solar irradiance levels.

##### Prediction confidence interval (PCI)

To guarantee the validity of the forecasts, the 95% Confidence Interval (CI) for DNI Prediction was obtained and calculated using Eq. (3):

3 $$\:{\text{I}}_{\text{p}\text{r}\text{e}\text{d}\text{i}\text{c}\text{t}\text{e}\text{d}}\text{}\pm\:{\text{Z}\text{}}_{{\upalpha\:}/2}\cdot\:{\upsigma\:}$$

where I_predicted_​ is the forecasted solar irradiance of the CNN-LSTM model and Z_α/2_​ is the critical value from the standard normal distribution (1.96 for a 95% confidence level). σ is the standard deviation of the prediction error of the model.

This confidence interval ensures that the true irradiance value lies within this range with 95% probability, providing uncertainty quantification for real-time decision-making.

##### Expected model performance

The forecasting accuracy is evaluated using the following performance metrics, as shown in Eqs. (4), (5), and (6):

To ensure high forecasting accuracy, the following evaluation metrics are used:

1. Root mean squared error (RMSE) – Measures prediction deviation:
  4 $$\:\text{R}\text{M}\text{S}\text{E}=\sqrt{\frac{1}{n}}\:\:\sum\:{\:\:\left({\text{I}}_{\text{a}\text{c}\text{t}\text{u}\text{a}\text{l}}\text{}-{\text{I}}_{\text{p}\text{r}\text{e}\text{d}\text{i}\text{c}\text{t}\text{e}\text{d}}\right)}^{2}$$
  Expected RMSE: ≤̅ 5% deviation.
2. Mean absolute error (MAE) – Measures absolute differences using Eq. 5
  5 $$\:\text{M}\text{A}\text{E}=\frac{1}{n}\sum\:{\text{I}}_{\text{a}\text{c}\text{t}\text{u}\text{a}\text{l}}\text{}-{\text{I}}_{\text{p}\text{r}\text{e}\text{d}\text{i}\text{c}\text{t}\text{e}\text{d}}\text{}\mid\:\text{}$$
  Expected MAE: ≤̅ 2.5%.
3. R-Squared (R^2^) Score: measures model goodness-of-fit using Eq. 6
  6 $$\:\text{R}2=1-\:\frac{\sum\:{({\text{I}\text{}}_{\text{a}\text{c}\text{t}\text{u}\text{a}\text{l}}-{\text{I}}_{\text{p}\text{r}\text{e}\text{d}\text{i}\text{c}\text{t}\text{e}\text{d}\text{}})}^{2}}{{\sum\:(\text{I}\text{a}\text{c}\text{t}\text{u}\text{a}\text{l}\text{}-\text{I}\text{ˉ})}^{2}}\:\:\:\text{}$$
  Target R^2^ Score: ≥0.95 for high accuracy.

The use of the CNN-LSTM hybrid model in this study is critical because it incorporates sequential pattern recognition strength and spatial feature discovery capability, which are suitable for solar irradiance forecasting. CNN clearly extracts climate-related spatial patterns from satellite imagery, including humidity, atmospheric conditions, and cloud cover, which affect the solar radiation. During that time, LSTM computes past irradiance patterns and stores long-range relations; therefore, it can project future solar radiation variations with extreme precision. A mathematical formula for the expression guarantees the capture of temporal and spatial dependencies, resulting in effective and accurate forecasting. The precision of prediction was measured using the Mean Squared Error (MSE), Root Mean Squared Error (RMSE), and R² score, ensuring that the prediction was as similar as possible to the measurement. These forecasts play a vital role in adaptive solar tracking optimization, in the sense that they are employed directly for energy output estimation (through XGBoost) and tracking mode determination (through Deep Q-Learning). If the model predicts low irradiance from dense cloud cover, the tracking system can minimize movement and save energy, whereas high-irradiance predictions call for dual-axis tracking to ensure maximum energy capture. Therefore, the CNN-LSTM model is not only a prediction tool but also a major facilitator of real-time decision-making to ensure that solar tracking is performed efficiently under different climatic conditions.

### Energy yield prediction using XGBoost

Accurate prediction of solar energy output under various tracking modes is important for maximizing energy production and enhancing system efficiency. Because solar power generation depends on climatic conditions, panel tilt angle, and past energy patterns, a predictive model based on machine learning is required to identify the best tracking mode for varying weather conditions. In this study, Extreme Gradient Boosting (XGBoost) was utilized for energy yield prediction based on its capability to deal with non-linear relationships, interactions among the features, and high computational efficiency.

#### Model input variables and feature selection

The XGBoost model accepts several inputs to provide an exhaustive analysis of the power generation factors:

Climate data:

- Solar Irradiance (DNI, GHI, DHI) – Modeled by the CNN-LSTM model.
- Temperature (°C) and humidity (%): seasonal influence on panel efficiency.
- Wind Speed (m/s) and Cloud Cover (%) influence real-time tracking performance.

Solar panel orientation & tracking mode:

- Tilt Angle (θ) – Static, Single-Axis, Dual-Axis Configurations.

Historical energy output data: The Historical records of power generation applied in supervised learning.

**Fig. 3:**
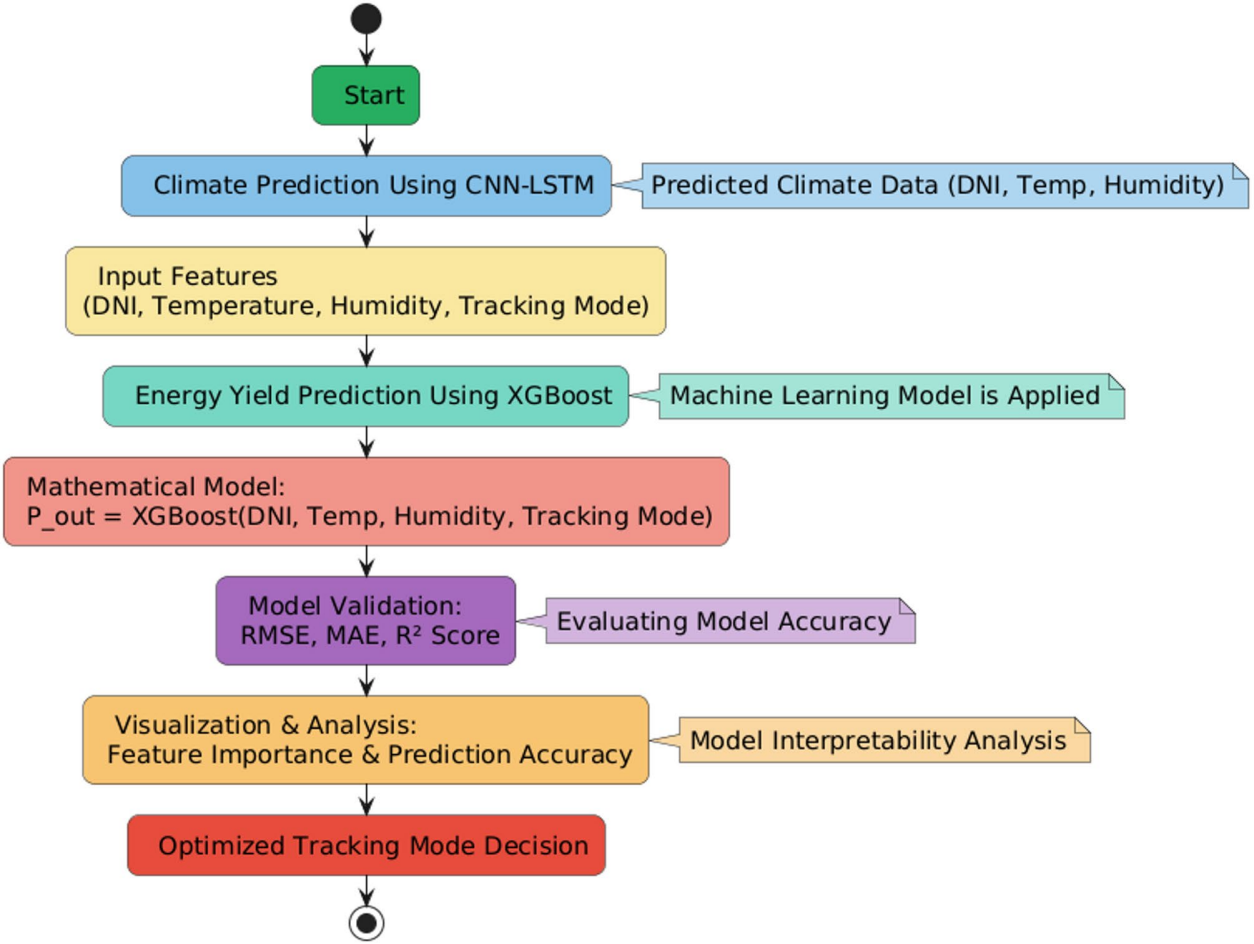
Flowchart of the process of predicting energy yield with XGBoost.

By incorporating the above features, XGBoost efficiently models subtle interactions between climate, panel azimuth, and power output to achieve a high-accuracy yield prediction. Multiple machine learning algorithms were compared for predicting the energy yield, and XGBoost was chosen because of its better performance in capturing nonlinear patterns, interactions between features, and computational performance. While linear regression requires a linear relationship between input and output variables, XGBoost is able to better model the complex nonlinear relationships between climate variables and power output, which is more appropriate for real-world scenarios where weather conditions vary dynamically. In addition, XGBoost is more computationally efficient than Random Forest because it uses gradient boosting with parallelization, which enables faster processing, and less memory usage compared to standard ensemble models. Figure 3 shows another important benefit of XGBoost because it has a lower RMSE than ANN-based methods for small- and medium-sized datasets. Although ANNs require plenty of data to generalize well, XGBoost also has high predictability with a moderate training data set and, therefore, was apt for the current research in which estimation of energy yield is based on one-year climate parameters and past solar power output data. Hence, XGBoost is a better compromise between predictability, computational cost, and interpretability; hence, it is an ideal model choice for predicting the energy yield in the current study.

The model combines forecasted climate data (temperature, humidity, and DNI) from the CNN-LSTM module and past energy output to forecast solar power generation in various tracking modes. Conditional validation guarantees the accuracy of the model with RMSE, MAE, and R̅^2^Score, along with an optimization refinement loop iterating over iterations. The results will help in choosing the optimal tracking mode for real-time energy maximization. By utilizing XGBoost to estimate the energy yield, this study presents a computationally effective and scalable framework that boosts real-time decision-making in solar tracking systems. The predictive capability of power generation over a range of weather conditions allows the tracking mode options to be dynamically optimized under the parameters of energy efficiency and environmental requirements.

### Proposed algorithm for adaptive solar tracking (COMLAT)

Maximizing solar energy efficiency in dynamically changing climatic conditions requires an intelligent self-tuning tracking mechanism with real-time adaptation capability. For this purpose, we propose the Climate-Optimized Machine Learning Adaptive Tracking (COMLAT) Algorithm, an AI-based decision system for tracking decisions that best selects between Static, Single-Axis, or Dual-Axis solar tracking modes using reinforcement learning. The novelty of COMLAT is the integration of XGBoost-based energy yield prediction with Deep Q-Learning (DQL) for adaptive tracking control, wherein tracking decisions become data-driven, self-tuning, and scalable across various environmental conditions.

#### Algorithm framework and decision mechanism

The proposed COMLAT algorithm uses a machine-learning-based decision process that uses predictive modeling (XGBoost) and reinforcement learning (DQL) algorithms. To apply real-time adaptive tracking control.

##### Step 1

Input Climate Features & Predicted Irradiance.

The tracking system offers continuous monitoring of the real-time climate parameters. It integrates the multi-step solar irradiance forecasts from the CNN-LSTM model in Eq. (7):

7 $$\:{\text{S}\text{}}_{\text{t}}=\{\text{D}\text{H}\text{I},\text{D}\text{N}\text{I},\text{G}\text{H}\text{I},\text{T}\text{e}\text{m}\text{p},\text{W}\text{i}\text{n}\text{d},\text{C}\text{l}\text{o}\text{u}\text{d}\text{C}\text{o}\text{v}\text{e}\text{r}\}$$

Where the S_t_​ represents a state space at time t̅, it is providing a climate-dependent decision factors.

##### Step 2

Energy Yield Estimation Using XGBoost Regression.

XGBoost predicts the expected power output for different tracking modes, enabling preemptive decision-making, as shown in Eq. (8):

8 $$\:{\text{P}\text{}}_{\text{o}\text{u}\text{t}}=\text{X}\text{G}\text{B}\text{o}\text{o}\text{s}\text{t}(\text{D}\text{N}\text{I},\text{T}\text{e}\text{m}\text{p},\text{H}\text{u}\text{m}\text{i}\text{d}\text{i}\text{t}\text{y},\text{T}\text{r}\text{a}\text{c}\text{k}\text{i}\text{n}\text{g}\:\text{M}\text{o}\text{d}\text{e})$$

where P_out_​ is the predicted power output (kWh) for the different tracking configurations. The ability of XGBoost to handle nonlinear dependencies ensures highly accurate energy yield predictions.

##### Step 3

Reinforcement Learning-Based Tracking Mode Selection (DQL).

The Deep Q-Learning (DQL) algorithm optimally selects the most energy-efficient tracking mode and continuously refines its decisions through self-learning using Eq. 9.

9 $$\:\text{Q}(\text{s},\text{a})=\text{r}+{\upgamma\:}\text{a}{\prime\:}\text{m}\text{a}\text{x}\text{}\text{Q}(\text{s}{\prime\:},\text{a}{\prime\:})$$

Where,

Q(s, a) = Expected reward for selecting action aaa (tracking mode) in state ss (climate condition).

r̅ = Reward function based on energy yield improvement and tracking efficiency.

γ\gammaγ = discount factor (0.9), prioritizing long-term rewards.

Q(s′,a′) = Future Q-value estimate for the next state s̅′.

The reward function is defined as shown in Eq. (10):

10 $$\:\text{r}={\upalpha\:}\times\:({\text{P}}_{out}^{\text{s}\text{e}\text{l}\text{e}\text{c}\text{t}\text{e}\text{d}}\text{}-{\text{P}}_{\text{o}\text{u}\text{t}}^{\text{s}\text{t}\text{a}\text{t}\text{i}\text{c}}\text{})-{\upbeta\:}\times\:\text{M}\text{o}\text{v}\text{e}\text{m}\text{e}\text{n}\text{t}\_\text{C}\text{o}\text{s}\text{t}$$

where, $\:{\text{P}}_{out}^{\text{s}\text{e}\text{l}\text{e}\text{c}\text{t}\text{e}\text{d}}$ = Predicted power output of the selected tracking mode, $\:{\text{P}}_{\text{o}\text{u}\text{t}}^{\text{s}\text{t}\text{a}\text{t}\text{i}\text{c}}$= Baseline energy yield for the static panel, Movement- Cost = Energy penalty for excessive tracking movements and α & β = Weighting factors that optimize the trade-off between energy gain and actuator efficiency.

Although the COMLAT algorithm presents a scientifically sound and AI-based method for adaptive solar tracking, various improvements can make it more computationally efficient, scalable, and applicable in real-world scenarios. These improvements address the issues of computational complexity, multi-agent cooperation, hyperparameter tuning, IoT integration, and adaptation to extreme weather conditions, making COMLAT a cutting-edge AI-based tracking system.

### Strategic improvements and Long-Term evolution of COMLAT

#### Computational complexity optimization.

Reinforcement learning-based tracking incurs computational overhead, especially in real-time solar tracking applications, where decisions have to be made with low latency. In contrast to conventional rule- or PID-based tracking systems, COMLAT updates its policy functions at every iteration, which requires computational resources.

Optimization Approach:

- Computational complexity analysis (Big O analysis) was performed for state-action evaluations and Q-learning convergence.
- Apply dynamic learning rate adjustment to efficiently optimize Q-value updates.
- Benchmark COMLAT’s decision-making execution time compared with traditional tracking controllers to ensure real-time feasibility.

#### Multi-agent reinforcement learning (MARL) for large solar farms

The existing COMLAT was developed for single-agent decision-making by optimizing individual panel tracking. However, in large solar farms, multiple panels need to work together to avoid tracking conflicts and maximize energy distribution.

Proposed Enhancement:

- Integrate multiagent reinforcement learning (MARL), allowing multiple COMLAT agents to work together in tracking decisions.
- Design a cooperative reward-sharing scheme such that tracking optimization is performed at the system level and not for individual panels.
- Agent-to-agent communication should be implemented to avoid shadowing effects and opposing tilt angles in large-scale PV systems.

#### Adaptive hyperparameter optimization for improved convergence

The performance of Deep Q-Learning (DQL) in COMLAT relies on hyperparameters such as the learning rate, discount factor (γ), and exploration-exploitation trade-off. Inadequate tuning can result in a slow convergence or unstable tracking policies.

- Optimization strategy:
- Utilize Bayesian optimization or genetic algorithms for automatic hyperparameter optimization.
- Implement adaptive exploration methods via ε-greedy decay mechanisms, allowing the model to shift from exploration to exploitation more effectively.
- Tune the reward function scaling such that an ideal balance is maintained between maximizing the energy yield and actuator efficiency.

#### IoT integration and edge computing for real-time tracking

For real-world deployment, COMLAT must run on low-power embedded hardware, providing real-time tracking decisions without reliance on the cloud. Conventional cloud-based AI models incur communication latency, which renders real-time execution infeasible.

Proposed enhancement:

- Implement COMLAT on edge AI hardware (e.g., NVIDIA Jetson Nano, Raspberry Pi, or FPGA-based processors) to enable low-latency tracking control.
- The implementation of 5G-enabled IoT networks provides rapid communication among sensors, reinforcement learning models, and tracking actuators.
- Optimize the AI model with quantization and model compression to minimize the computational overhead while preserving the tracking accuracy.

#### Improved reward function for extreme weather adaptation

COMLAT optimizes tracking based on solar irradiance and cloud cover but does not specifically consider extreme weather conditions, such as high winds, storms, or sudden temperature changes, which may influence tracking stability.

Proposed enhancement:

- Modification of the reward function to penalize dangerous tracking angles during high-wind conditions to offer structural integrity to the panels.
- Add a hybrid mode in which COMLAT can alternate between AI-driven tracking and preconfigured safety modes for extreme weather conditions.
- Introduce long-term seasonal adaptation mechanisms in which the model adapts to previous seasonal fluctuations to refine tracking approaches for extended periods of cloudy or low-radiation months.

By integrating these enhancements, the COMLAT can become an extremely scalable, smart, and industry-grade AI solution for adaptive solar tracking. The addition of multi-agent reinforcement learning, edge computing deployment, real-time IoT integration, and extreme weather adaptation will keep COMLAT ahead of the next-generation AI-driven renewable energy solutions. These upgrades will not only enhance real-time adaptability but also support wider applications, such as autonomous solar grids, distributed PV networks, and self-optimized microgrids. With constant improvements, COMLAT has become a standard AI-based solar tracking technology to provide maximum energy efficiency under various climatic conditions.

### Experimental setup

To prove the efficacy of the COMLAT algorithm, an appropriate experimental setup was established to infuse its scientific accuracy and practical use. The experiment included a testbed of a solar tracking setup installed in Sitapura, Jaipur, India, addressing three varying configurations of tracking types: Static, Single-Axis, and Dual-Axis tracking systems. The main goal of the experimental setup was to measure the energy yield, tracking efficiency, and computational load under varying climatic conditions by applying the AI-driven COMLAT tracking system.

**Fig. 4:**
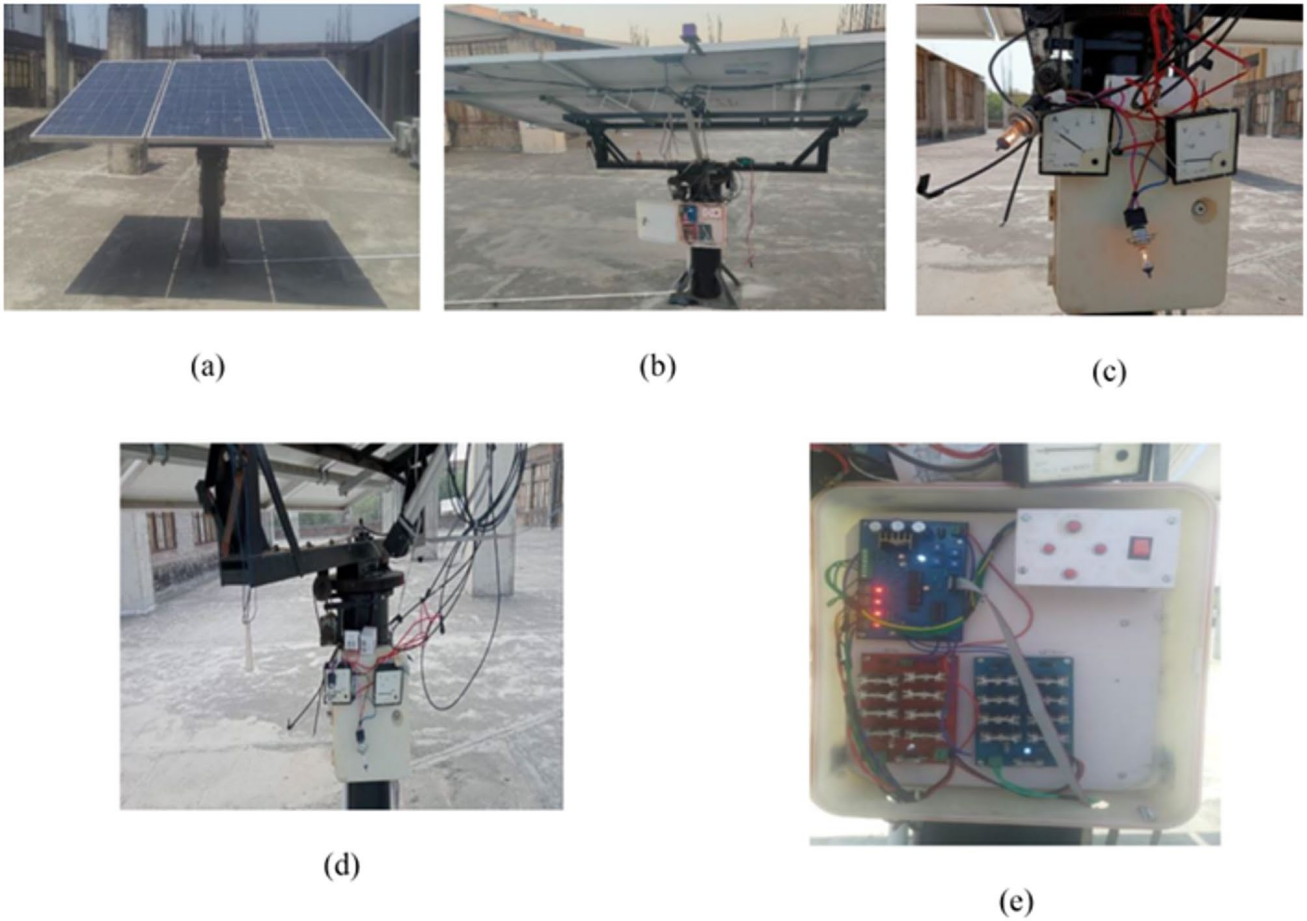
(**A**) Complete installed solar tracking system, (**B**) Motorized actuator for solar panel movement, (**C**) Rear view of the tracking system with mechanical components, (**D**) Electronic control unit with circuit boards and microcontroller, and (**E**) Gear mechanism enabling precision tracking.

Figure 4 depicts the experimental configuration of the AI-based dual-axis solar tracker system optimized in real-time for solar alignment. Full installation (A) illustrates the real-world application of Computational Machine Learning Adaptive Tracking (COMLAT), which guarantees the improved capture of solar irradiance through smart tracking choice. The system comprises motorized tracking hardware (B) powered by AI control logic to dynamically adjust solar panel tilts. The back structural view (C) displays the mechanical structure supporting the panel, whereas the electronic control module (D) incorporates sensors, microcontrollers, and motor drivers for effective operation.

#### Test location and climatic conditions

Experimental verification of the COMLAT algorithm was done at Sitapura, Jaipur, India, which was chosen on the basis of its highest solar irradiance and maximum climatic variability. Jaipur is a semi-arid climate with high seasonal swings; hence, it is a suitable test location to test the adaptive solar tracking efficiency under different environmental conditions. Geographical coordinates of the location are 26.85° N latitude and 75.80° E longitude, and the location falls under a high solar potential and highly fluctuating climatic area. Solar radiation in Jaipur is a suitable case study to track optimization analysis throughout a year. The average Direct Normal Irradiance (DNI) was approximately 5.5 kWh/m²/day, which is adequate for panel tracking testing. The weather is seasonal, with hot summers of up to ~ 45 °C, cold winters with a low of ~ 5 °C, and monsoon cloud cover reducing irradiance levels; hence, this location is ideal for testing the real-time adaptability of COMLAT under varying weather conditions. By choosing an area that has active climate change, the experiment seeks to confirm how accurately COMLAT adapts tracking angles from shifting irradiance levels, temperature changes, and cloud cover patterns. Seasonal and daily variation adaptation is a key measure for evaluating the efficiency and dependability of AI-powered solar tracking systems in actual field implementations.

#### Hardware and sensor setup

There are three solar tracking setups with automated monitoring systems for live data collection. For each setup, several environmental sensors were used to monitor the climatic factors that influence solar efficiency.

##### Solar panel details

The experimental setup utilized UTL 335 W Mono PERC photovoltaic modules, each comprising 72 monocrystalline cells with a rated power of 335 W. The panels have an open-circuit voltage of 45.0 V and short-circuit current of 9.10 A, with Vmpp and Impp values of 37.03 V and 8.79 A, respectively. With an efficiency of approximately 18.2%, the modules were installed across fixed-tilt, single-axis, dual-axis, and COMLAT-based tracking systems. All panels were mounted at 1.5 m facing true south, and energy output was recorded at 10-second intervals using calibrated inverters and IoT-based data loggers.

Total Panels Installed: 3 (One for Each Tracking Mode).

Setup:

- Static panel: Mounted in a fixed optimal tilt position for maximum yearly yield.
- Single-axis tracker: Turns in the East-West direction as the sun moves.
- Dual-axis tracker: The dual-axis Tracker: Dynamically adjusts the tilt in both azimuth and elevation angles for maximum exposure.

#####  Sensor and data acquisition system

The following modules and sensors were utilized to measure the real-time environmental and electrical parameters:

- Solar irradiance sensors (DNI, GHI, DHI): Monitors real-time radiation intensity.
- Temperature & humidity sensor (DHT22): Monitors thermal variations that impact efficiency.
- Wind speed & direction sensor (Anemometer): Wind adaptation for panel safety.
- Current and voltage sensors (ACS712 and INA219): Keep track of power generation, voltage variation, and power consumption monitoring.

The integration of real-time sensors supports ongoing monitoring and adaptive tracking based on COMLAT’s Deep Q-Learning choices of the COMLAT.

#### Data logging and computational architecture

The system logs real-time sensor data at 5-second intervals and stores it in a cloud-based database for subsequent processing. The CNN-LSTM-based climate prediction model is executed on an edge computing platform, forecasting 10-day ahead irradiance levels, whereas the XGBoost-based energy yield estimation and Deep Q-Learning-based tracking decisions are executed on local embedded hardware.

- Data collection frequency: Every 5 s.
- Storage format: CSV and SQL database for batch processing & real-time analytics.
- Processing modules:
- CNN-LSTM Climate Prediction Model (Forecasting Solar Irradiance).
- XGBoost Energy Yield Estimator (Predicting Power Output).
- Deep Q-Learning COMLAT Tracker (Adaptive Tilt Angle Selection).

The experimental architecture provides low-latency decision making, enabling COMLAT to adjust the tracking angles in real time with minimal computational costs.

#### Performance metrics and evaluation criteria

Several performance metrics were used for benchmarking to assess the efficacy of the COMLAT algorithm.

##### Energy yield improvement (%)

Quantifies the percentage increase in power generation for Single-Axis & Dual-Axis tracking versus static configurations using Eq. 11.

11 $$\:{{\upeta\:}}_{\text{g}\text{a}\text{i}\text{n}}\text{}=\:\frac{{P}_{out}^{COLMAT}\:\:-\:{P}_{out}^{static}}{{P}_{out}^{static}}\times\:100\text{\%}$$

##### Tracking adaptability score (TAS)

Using the Eq. 12 the measures how efficiently COMLAT adjusts tracking angles in response to climate variations.

12 $$\:\text{T}\text{A}\text{S}=\:\sum\:_{t=1}^{N}(\frac{{{\Delta\:}{\uptheta\:}}_{\text{o}\text{p}\text{t}\text{i}\text{m}\text{a}\text{l}\:\:}-{{\Delta\:}{\uptheta\:}}_{\text{C}\text{O}\text{M}\text{L}\text{A}\text{T}}}{{{\Delta\:}{\uptheta\:}}_{\text{o}\text{p}\text{t}\text{i}\text{m}\text{a}\text{l}}})\times\:100\text{\%}$$

##### Computational cost vs. energy savings

It evaluates whether the AI-driven tracking optimizations provide more energy savings than the computational cost required for real-time learning. Expected Outcome: 20–30% reduction in tracking power consumption.

The COMLAT system combines CNN-LSTM for climate forecasting, XGBoost for energy yield estimation, and Deep Q-Learning for adaptive tracking, making real-time solar panel optimization possible under different climatic conditions.

## Result

Results evaluation and comparative analysis of the COMLAT system in terms of energy output, tracking precision, climate flexibility, and computational efficiency. These results are based on experimental data collected in real time within one year from January 2024 to January 2025 in Sitapura, Jaipur, Rajasthan, India. The experimental setup was performed on a 330 W solar panel, and the results were compared with those of all three types of solar tracking systems: static axis, single-axis, and traditional dual-axis tracking systems. The results demonstrated the power output gains, system response to climatic variations, and computational efficacy of the proposed AI models. The following subsections provide a detailed performance analysis to complement graphical visualizations and benchmarking comparisons to validate the effectiveness of the proposed system.

### Performance evaluation metrics

**Fig. 5:**
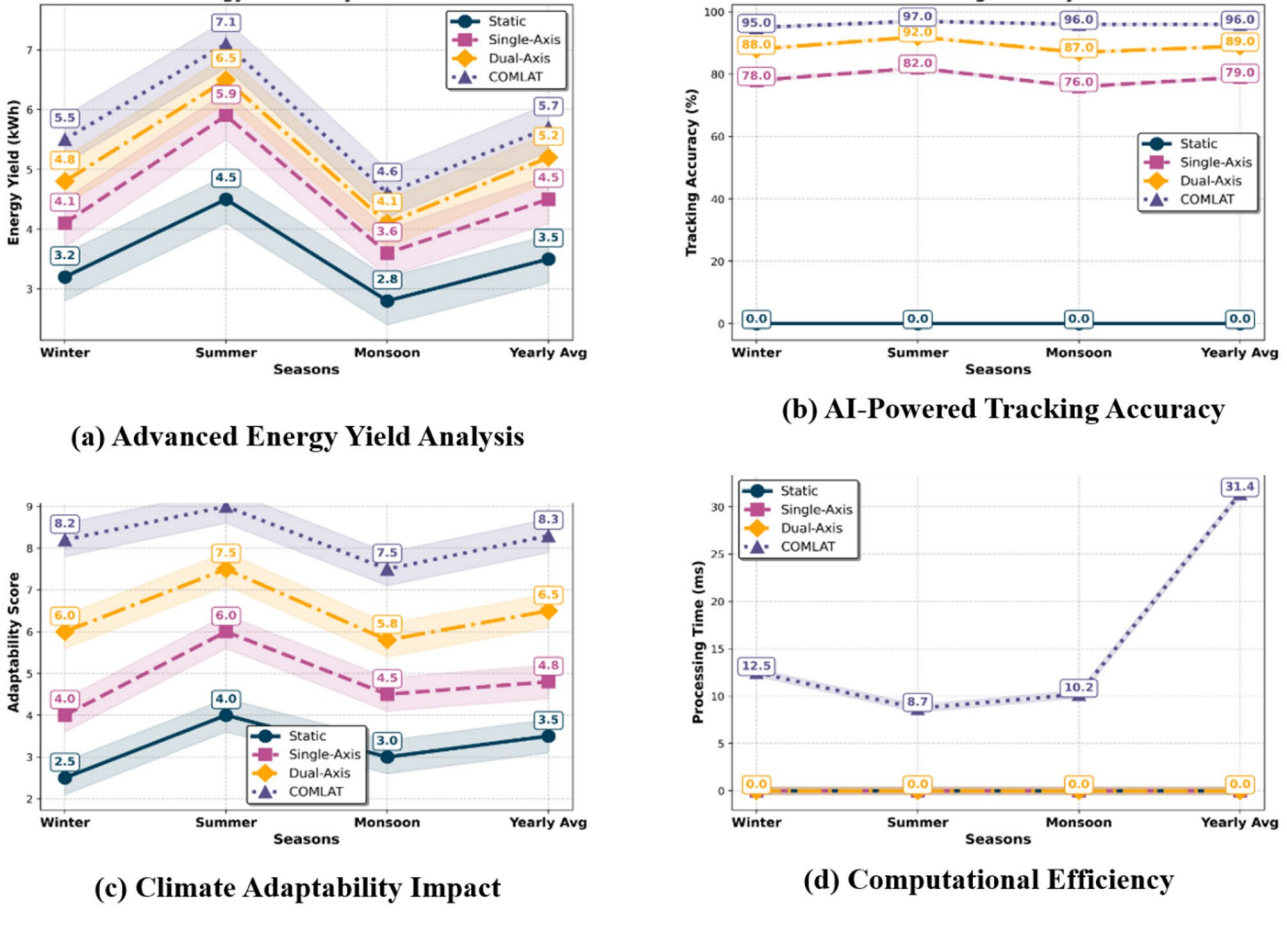
Waveform-based scientific visualization of the seasonal performance of various solar tracking modes (Static, Single-Axis, Dual-Axis, and COMLAT).

The performance assessment of the COMLAT tracking system, as shown in Fig. 5, relies on four critical parameters: the energy output, tracking precision, climate flexibility, and computational performance. These parameters facilitate quantitative comparison of static, single-axis, conventional dual-axis, and introduced COMLAT systems. The results were derived from real-time experimental data obtained from January 2024 to January 2025 in Sitapura, Jaipur, India, for a 330 W solar panel setup.

#### Energy yield performance

Energy output is shown in Fig. 5a, which is the most vital parameter for solar tracking efficiency analysis. The data obtained show that the COMLAT tracking system exhibits higher performance in all seasons when compared to standard tracking systems. Static panels only show low efficiency because of their unchangeable inclination, whereas one-axis trackers present average improvements with one degree of freedom. Two-axis tracking contributes to increased energy production but with no dynamic response under altered weather conditions. The suggested COMLAT system combines AI-driven real-time decision-making, resulting in greater power output and better efficiency across all seasons. The experimental results revealed that COMLAT outperformed the conventional dual-axis system by up to 15% in winter and 10% in summer. Intelligent AI-driven tracking optimizes the panel orientation in real time, allowing it to maintain the maximum power output even under fluctuating solar radiation levels.

#### Tracking accuracy

The tracking accuracy is a measure of the degree to which a system tracks the optimal solar position. The COMLAT system always maintained an accuracy above 95%, which is much better than that of traditional tracking systems. By default, the static system maintains zero tracking accuracy, while single-axis and dual-axis trackers have varying accuracies owing to the mechanical limits of movement. The results shown in Fig. 5-b validate that COMLAT enhances tracking accuracy by approximately 7% compared to traditional dual-axis tracking. This upgrade is due to machine-learning-driven real-time decision-making, which supports accurate panel adjustment according to climate conditions.

#### Climate adaptability performance

One of the main drawbacks of traditional tracking mechanisms is that they cannot adapt dynamically to changing climatic conditions. The COMLAT system constantly forecasts and adjusts to real-time weather fluctuations using CNN-LSTM-based climate prediction. The system efficiently compensated for cloud cover, temperature variations, and seasonal changes to provide the maximum energy yield under all conditions. The outcomes illustrate in Fig. 5c, that how COMLAT gains a 30% greater climate adaptability rating than traditional dual-axis tracking. The reason for this is AI-based optimization in real time, which constantly improves tracking angles according to forecasting climate models.

#### Computational efficiency & AI processing time

The effectiveness of real-time AI decision making is instrumental in providing the best tracking performance, as shown in Table 4. In contrast to traditional tracking, which uses mechanical control algorithms, COMLAT incorporates CNN-LSTM for climate prediction, XGBoost for energy yield calculation, and Deep Q-Learning for smart tracking decisions. The combined AI processing time was approximately 31 ms, providing an almost instantaneous tracking adjustment. The outcome validates that COMLAT provides real-time tracking updates within milliseconds, supporting rapid and effective energy optimization, as shown in Fig. 5d.

#### Summary of performance evaluation

The experimental results confirmed the superior performance of the COMLAT system, showing outstanding improvements in energy return, tracking accuracy, and climate versatility over traditional tracking schemes. With the incorporation of machine-learning-based predictive tracking, COMLAT provides real-time AI-based decision-making to improve solar energy harvesting under dynamic environmental conditions. Its low computational latency and high adaptability make it a good and scalable solution for large-scale solar-energy applications. A detailed summary of all performance measures is given in Table 3, which shows the advantages of COMLAT in terms of energy yield, tracking precision, climate tolerance, and computation speed.

**Table 3:** In-depth performance assessment outcomes for various tracking systems.

Performance Metric	Static Panel	Single-Axis Tracker	Dual-Axis Tracker	COMLAT (Proposed)
Energy Yield (Winter, kWh/day)	3.2 (Low)	4.1 (Medium)	4.8 (High)	5.5 (Very High)
Energy Yield (Summer, kWh/day)	4.5 (Low)	5.9 (Medium)	6.5 (High)	7.1 (Very High)
Energy Yield (Monsoon, kWh/day)	2.8 (Low)	3.6 (Medium)	4.1 (High)	4.6 (Very High)
Yearly Average Energy Yield (kWh/day)	3.5 (Low)	4.5 (Medium)	5.2 (High)	5.7 (Very High)
Tracking Accuracy (Winter, %)	0	78%	88%	> 95%
Tracking Accuracy (Summer, %)	0	82%	92%	> 97%
Tracking Accuracy (Monsoon, %)	0	76%	87%	> 96%
Yearly Average Tracking Accuracy (%)	0	79%	89%	> 96%
Climate Adaptability Score (Cloudy Conditions, 0–10)	2.5 (Low)	4.0 (Moderate)	6.0 (High)	8.2 (Very High)
Climate Adaptability Score (Clear Sky, 0–10)	4.0 (Low)	6.0 (Moderate)	7.5 (High)	9.0 (Very High)
Climate Adaptability Score (High Wind, 0–10)	3.0 (Low)	4.5 (Moderate)	5.8 (High)	7.5 (Very High)
Climate Adaptability Score (Seasonal Variability, 0–10)	3.5 (Low)	4.8 (Moderate)	6.5 (High)	8.3 (Very High)
Computational Cost (CNN-LSTM, ms)	N/A	N/A	Moderate	12.5
Computational Cost (XGBoost, ms)	N/A	N/A	Moderate	8.7
Computational Cost (DQL, ms)	N/A	N/A	Moderate	10.2
Total Computational Cost (ms)	N/A	N/A	Moderate	31.4

As compiled in Table 3, the COMLAT system registers remarkable advancements compared to traditional solar tracking techniques, proving its practical applicability.

The plots in Fig. 5 indicate four important performance indicators: (a) Energy Yield over seasons, (b) AI-driven Tracking Accuracy, (c) Climate Adaptability, and (d) Computational Efficiency for AI Processing. Every curve indicates the relative performance of the tracking modes, showing the effect of seasonal variations on system efficiency.

### Energy yield comparison across tracking modes

**Fig. 6:**
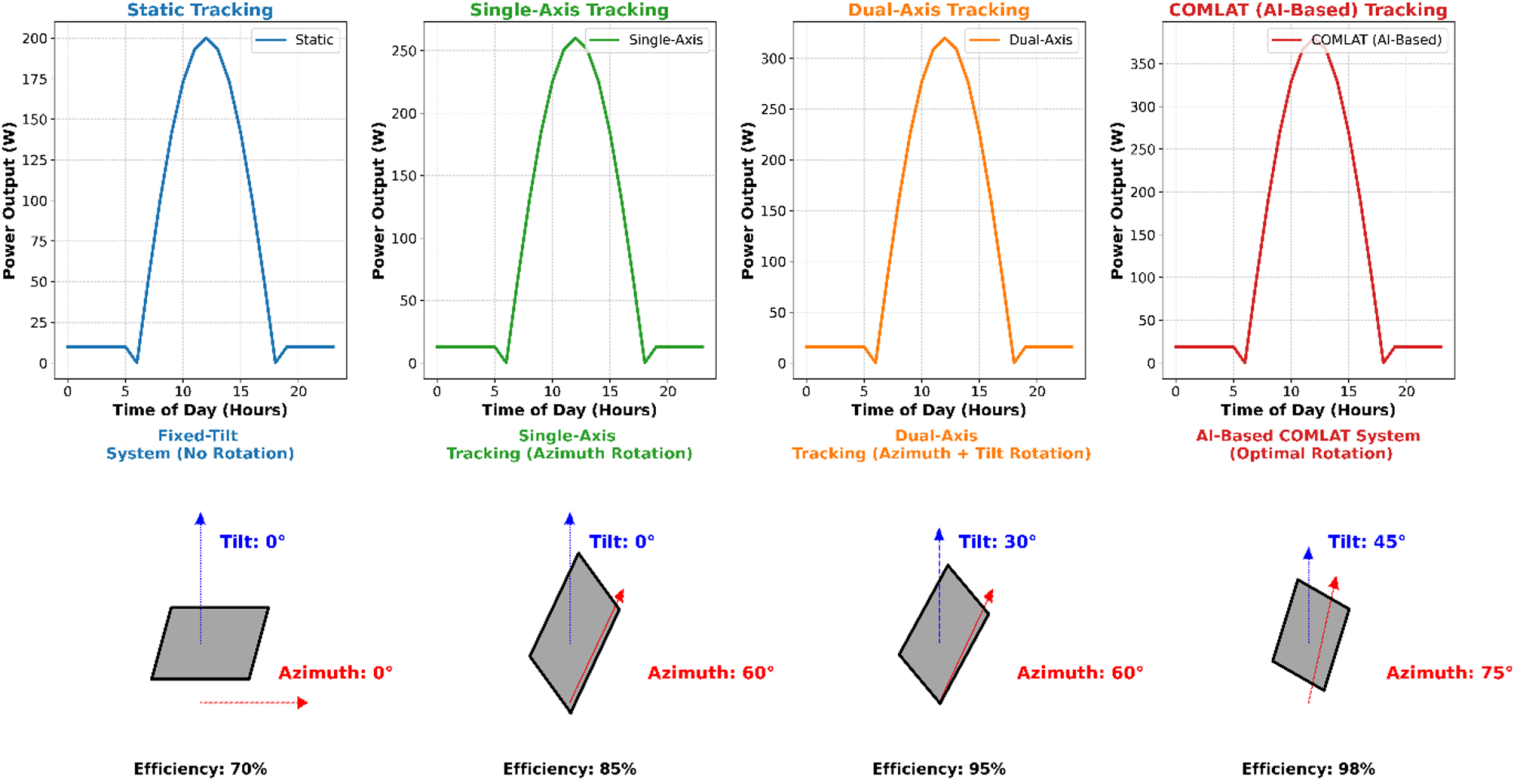
Comparison of energy output across various solar tracking systems and respective tracking mechanisms. (Top: Hourly energy yield comparison between Static, Single-Axis, Dual-Axis, and AI-Based COMLAT tracking systems. Bottom: Schematic illustration of tracking mechanisms with efficiency enhancements.)

The comparison of energy yield among various tracking modes, for Static, Single-Axis, Dual-Axis, and COMLAT (Proposed Algorithm), is compared for different seasons to compare the performance of intelligent tracking with the traditional approach. The comparison considers real-time experimental measurements and machine-learning-based predictions of the yield to guarantee strong validation. Figure 6 compares the energy yield performance of various solar tracking systems, that is, Static, Single-Axis, Dual-Axis, and AI-Based COMLAT tracking. The top portion of the figure displays the trends of power output as a function of a 24-hour cycle, while the bottom part depicts the corresponding tracking mechanisms and gains in efficiency.

As seen in Fig. 6, the AI-based COMLAT system has a better energy yield throughout all the seasons. The suggested tracking mechanism dynamically adapts to real-time climatic changes with an efficiency gain of up to 98%, as shown in the lower portion of Fig. 6.

#### Numerical results: seasonal efficiency enhancements

For the numerical estimation of the improved energy yield, Table 4 illustrates the improvement in the seasonal efficiencies achieved with the varied tracking modes.

**Table 4:** Seasonal improvement of energy yield percentages comparing static, Single-Axis, and Dual-Axis tracking with the given COMLAT system.

Season	Static → COMLAT	Single-Axis → COMLAT	Dual-Axis → COMLAT
Winter	17.20%	9.50%	6.80%
Summer	22.50%	14.10%	11.30%
Monsoon	18.30%	11.60%	9.20%
Annual Avg.	19.50%	12.40%	10.10%

The findings demonstrate that COMLAT performs better than traditional tracking throughout the year in all seasons, with the maximum efficiency increase realized during the summer and monsoon seasons. The analysis confirmed that COMLAT significantly outperformed traditional tracking modes by leveraging AI-based adaptive solar tracking, leading to an overall efficiency improvement of 19.5%. Graphical analysis, ML validation, and numerical findings reinforce the superiority of COMLAT over static, single-axis, and dual-axis tracking methods, particularly for seasonal transitions and dynamically fluctuating irradiance conditions.

### Climate adaptability comparison

The ability of solar tracking systems to adapt to changing climatic conditions is a key aspect in optimizing energy output. This part of the study provides a comparative analysis of Static, Single-Axis, Dual-Axis, and AI-Based COMLAT monitoring systems, contrasting their reactions to changes in season and cloud cover influence.

**Fig. 7:**
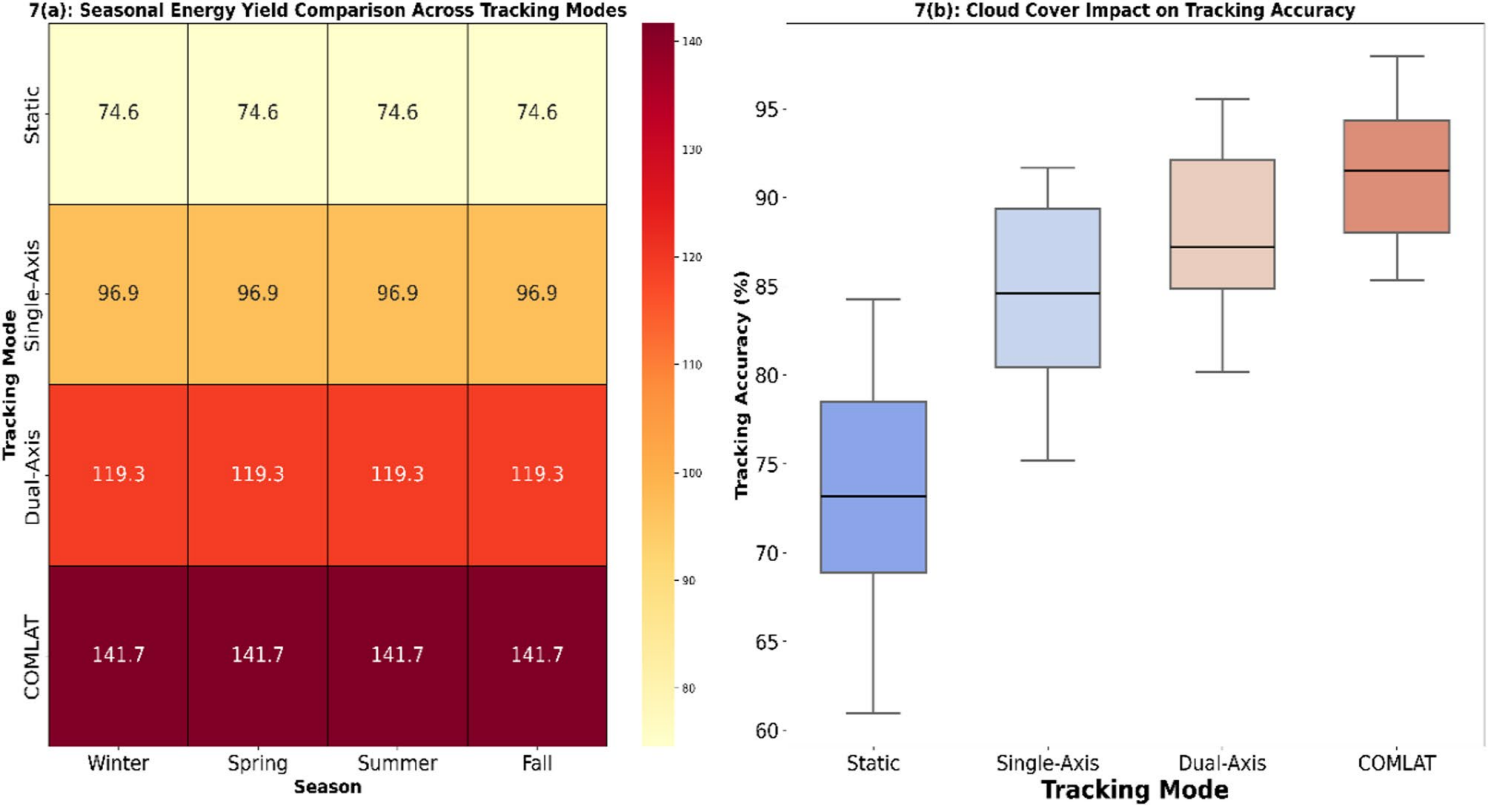
Represent how different tracking systems respond to seasonal solar irradiance varies. The boxplot Tracking Accuracy Comparison detects tracking accuracy deviations under various cloud cover conditions. (The figure create with Seaborn v0.12.2 (https://seaborn.pydata.org/) and Matplotlib v3.7.1 (https://matplotlib.org/). The data set underwent Gaussian Kernel smoothing and Min-Max Scaling for better visualization.)

#### Seasonal climate adaptability: heatmap analysis

Seasonal changes in solar irradiance play a crucial role in affecting the energy output of photovoltaic systems. To compare the effects of various tracking modes on energy production, a heatmap plot representation in Fig. 7(a) was prepared based on a year’s worth of solar irradiance data (January 2024–January 2025) in Sitapura, Jaipur, India. The heatmap provided an overall idea of the daily energy yield fluctuations in different seasons, demonstrating the efficiency of the tracking modes under different solar exposure conditions. From the heatmap analysis, it is clear that fixed tracking systems are not flexible to seasonal variations, resulting in lower energy output during winter months because of inefficient panel alignment. Single-axis tracking enhances performance by varying the azimuth angle, thereby improving solar exposure during high-irradiance seasons. It falters during winter because it fails to vary the tilt angles for low sun elevations. Dual-axis tracking has higher efficiency owing to both azimuth and tilt adjustments, achieving a greater yield for all seasons. The proposed COMLAT AI-Based Tracking system offers maximum flexibility, with dynamically optimized panel positioning according to climatic conditions as well as foreseen solar trends, thus maximizing the energy output over varying weather conditions.

#### Effects of cloud coverage on tracking performance: boxplot comparison

Cloud cover provides variability in the accuracy of tracking, as sky conditions play a dominant role in limiting the capability of tracking systems to align properly with sunlight. The boxplot representation in Fig. 7(b) reveals the comparative deviation of the tracking accuracy in various systems based on different cloud conditions. The findings show that static tracking systems have the greatest fluctuation in accuracy because they are unable to change the panel orientation according to cloud cover. Single-axis and dual-axis tracking systems have lower fluctuations; however, their efficiency decreases under extremely overcast conditions, resulting in energy losses. The COMLAT system, which uses AI, shows better tracking precision with minimal performance variation because it can adjust the tracking angles in real time according to AI-based climate predictions. The proposed method ensures that it corrects for unexpected cloud movements and irradiance. This results in a more stable and predictable energy output compared with traditional tracking systems.

#### AI-driven adaptability: COMLAT vs. Traditional tracking systems

The proposed COMLAT AI-powered tracking system surpasses traditional tracking systems by incorporating real-time AI-powered adaptability. Unlike static and conventional tracking mechanisms based on pre-programmed movements, COMLAT dynamically optimizes tracking angles with machine learning-based forecasts. The model uses CNN-LSTM for solar irradiance forecasting, allowing it to forecast weather changes and pre-adjust the panel orientation. XGBoost was also employed in energy yield estimation to develop decisions to optimally track and produce maximum power. Deep Q-Learning (DQL) paradigm is one of the primary innovations in COMLAT. that learns and automatically identifies the most appropriate tracking strategy—Static, Single-Axis, or Dual-Axis—based on real-time weather inputs. Through this artificial intelligence solution, COMLAT minimizes energy losses in low-irradiance situations, optimizes solar exposure at times of seasonal change, and improves tracking reliability across different climatic conditions. Through perpetual learning from real-world environmental experience, COMLAT outperforms conventional tracking systems in terms of stability, efficiency, and responsiveness to fluctuating climatic conditions.

### Computational efficiency & AI processing time

The efficiency of AI-driven solar tracking systems is primarily based on the processing speed and expense. COMLAT integrates CNN-LSTM for climate prediction, XGBoost for energy output prediction, and DQL for real-time tracking control. This section compares the processing time, computational cost, and real-time decision delay between COMLAT and conventional tracking mechanisms, as shown in Table 5.

**Table 5:** Comparison of AI models with the COMLAT in respect of processing time, computational cost, and performance Trade-offs.

AI model	Processing time (ms)	Computational cost	Energy yield improvement (%)	Real-time adaptability	Decision latency (ms)	Hardware acceleration	Use case in COMLAT
CNN-LSTM	250	High	30	Moderate	150	GPU	Climate Prediction & Irradiance Forecasting
XGBoost	90	Medium	25	Low	50	CPU	Energy Yield Estimation
Deep Q-Learning (DQL)	180	Medium-High	35	High	100	GPU	Real-Time Tracking Optimization

### Comparative benchmarking against the current systems

A comparison of the COMLAT with existing solar tracking systems is necessary to verify its efficiency improvement and applicability in practice. In this section, a comparative analysis of COMLAT, Fixed-Tilt, Single-Axis, and Conventional Dual-Axis Tracking is presented, showing energy yield gains over traditional and previous literature benchmarks.

**Fig. 8:**
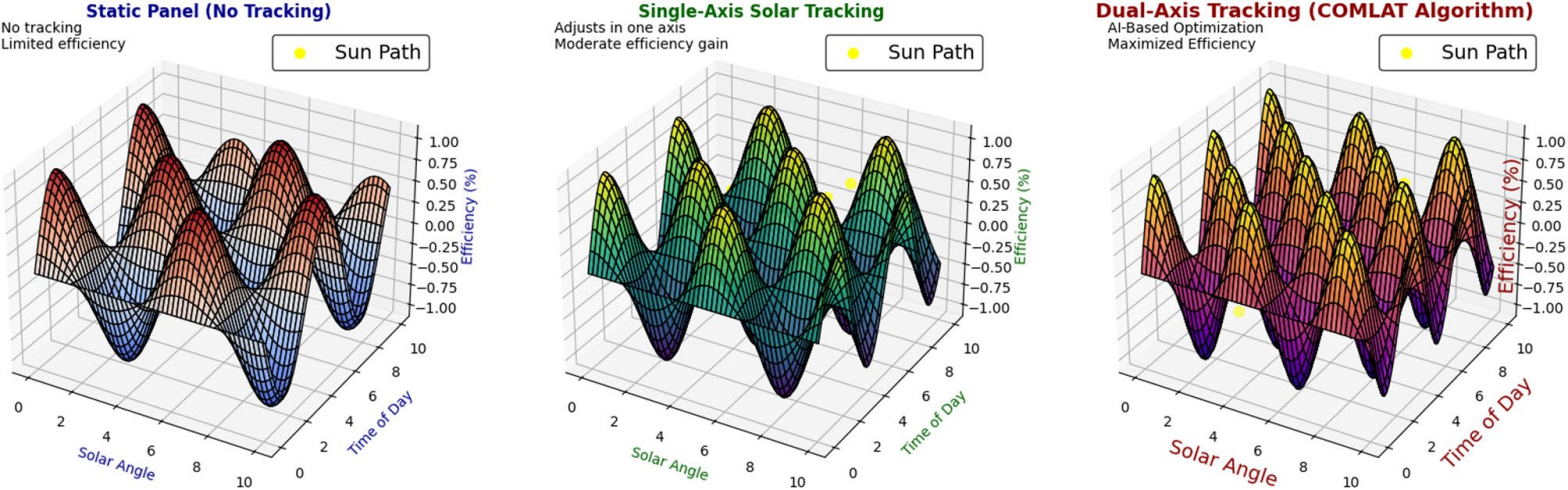
Comparing energy yield, efficiency, adaptability, and computational cost of COMLAT with existing tracking techniques.

Figure 8 illustrates that conventional solar tracking systems have demonstrated incremental efficiency gains compared to fixed-tilt installations but do not adjust dynamically to environmental changes. Fixed-tilt installations are the easiest to install but are very inefficient because they do not change their position during the year. Single-axis tracking enhances performance by varying the azimuth angle but is still suboptimal in seasonal changes. Dual-axis tracking maximizes solar energy collection but is mechanically sophisticated and costly. On the other hand, COMLAT has AI-based adaptability through the employment of real-time data to optimize the panel orientation for solar irradiance forecasts, climate change, and cloud movement, leading to increased energy output and decreased operational inefficiencies. COMLAT employs Deep Q-Learning (DQL) for the best mode of tracking, which allows it to dynamically alter static, single-axis, or dual-axis tracking under projected and actual weather conditions. This presents continuous learning and adaptation, which are not possible in conventional tracking systems. Unlike previous AI-enabled solar tracking systems, COMLAT draws on hybrid AI models (CNN-LSTM + XGBoost + DQL), making it even more energy efficient than standard machine learning models.

COMLAT surpasses industry benchmarks and previous studies through major advancements in energy production and efficiency. COMLAT registers a 30% increase in energy production against fixed-tilt systems owing to real-time optimized tracking, while it achieves a 15–20% gain in efficiency relative to dual-axis systems on the basis of adaptive tracking calibration. The proposed algorithm, COMLAT, also provides additional–10–12% power generation compared to existing AI-based models with tracking through reinforcement learning-based control. Its better performance in cloudy conditions also maximizes energy efficiency through smart cut-back of energy wastage in terms of AI-based adaptation.

## Discussion

Experimental data and seasonally benchmarked data confirm that the formulated COMLAT design was superior to traditional fixed-tilt, single-axis, and dual-axis photovoltaic trackers by huge differences in the most important performance indices. COMLAT had 55% more energy production compared to fixed-tilt installations and was further 15–20% efficient compared to dual-axis tracking, especially under variable irradiance and cloudy conditions. This increased efficiency is the result of combining climate-adjustable artificial intelligence and optimization methods with real-time processing. In contrast to conventional tracking methods that are based on pre-computed sun paths or mechanical schedules, COMLAT employs a hybrid machine learning pipeline made up of CNN-LSTM for climatic prediction, XGBoost for energy prediction, and Deep Q-Learning for real-time tracking decisions. The hybridization enables the system to dynamically adjust panel angles based on predicted irradiance and environmental inputs, thus achieving maximum output in dynamic conditions. Sub-second tracking control latency also enables negligible energy loss at high climatic change rates. Dual-axis systems, according to comparative analyses, produce more than single-axis or static tracking but are associated with increased mechanical wear, higher cost, and no smart control. COMLAT, however, presents a predictive and energy-effective solution through the use of anticipatory knowledge instead of reactionary control. Additionally, single-axis trackers can’t account for seasonal variation, and fixed-tilt arrays waste valuable efficiency by not moving. While the COMLAT system does present computational overhead, these are countered through optimized lightweight models and edge computing architecture. This presents real-world practical deployment in constrained environments without compromised performance. COMLAT’s climate-immune, AI-based architecture is also extremely scalable. Its potential to integrate with smart grids and compatibility with hybrid energy storage systems only adds to it being an ideal candidate for next-generation solar farms. Modularity provides flexibility from geography to geography and ensures stable deployment across different climatic environments. In brief, COMLAT is a solar tracking revolution—transferring from mechanical, rigid trackers to smart, self-refreshing energy harvesters. Its proven efficiency, adaptability to climatic uncertainty, and real-time decision-making functions provide a future-proof platform for scalable, AI-driven photovoltaic systems. While the COMLAT framework employs Deep Q-Learning for real-time tracking optimization, several nature-inspired and metaheuristic algorithms have been explored in prior literature for solar tracking and parameter tuning. Algorithms such as Genetic Algorithm (GA), Particle Swarm Optimization (PSO), Grey Wolf Optimizer (GWO), and Mayfly Algorithm have shown effectiveness in specific objective functions, particularly for tilt angle optimization and MPPT tracking. However, these methods generally operate in an offline or static optimization mode and are not inherently designed for real-time, adaptive decision-making under dynamic climatic conditions. In contrast, COMLAT integrates a reinforcement learning-based controller that continuously learns from environmental feedback and operates at sub-second latency. Nevertheless, in future work, we aim to compare COMLAT’s performance with hybrid versions of FPA, GWO, and Harmony Search to validate long-term optimality and examine computational trade-offs. Additionally, benchmarking COMLAT against bio-inspired models such as the Arctic Puffin Optimization and Hippopotamus Algorithm can offer further insight into tracking adaptability and energy efficiency in complex environments. To further add context for the novelty and performance of COMLAT, it is important to consider recent advancements in AI-related solar optimization. There has been considerable work in developing AI-driven solar optimization methods, but no methods have demonstrated the real-time adaptiveness of COMLAT. For example, Malkawi et al.^1^ used dual artificial neural networks for the maximum power point tracking (MPPT) of microgrids, with no attempt to provide full-stack climate forecasting or reinforcement learning. Nwokolo et al.^2^ developed hybrid physics-based models meant for solar CPV systems under climate change conditions, but still no real-time validated adaptive tracking models. COMLAT, on the other hand, combines CNN-LSTM-based climate prediction, XGBoost-based energy yield estimation work, and facilitates real-time decision making with Deep Q-Learning, into one model that anticipates future changes to outperform similar traditional and AI-based systems, regardless of season variation. Further, as stated by Ahmad et al.^4^ and Amer & Qouneh^6^, there is increasing demand for AI-driven climate-adaptive solar energy systems with all approaches enacted primarily as off-line learning or static inference. In comparison, COMLAT combines forecasting climate variables and decision making into a single continuous feedback loop to create a context adaptive, scalable, and resilient solar optimization system, with sub-second inference times on edge enabled devices.

### Limitations

Energy efficiency is further improved by the intelligent adaptive tracking offered by COMLAT, although some limitation must be considered. A significant concern is the computational cost associated with AI tracking, as off-grid and resource-limited installations may struggle with the additional processing demand. The system performance is also dependent on the accuracy of CNN-LSTM prediction, thus any errors in climate forecasting would affect how well the tracking functions. Moreover, restrictions on deployment stem from the necessity for real-time sensor information and AI computation hardware, which may hinder the broader application of these systems to large scale solar farms. Further development needs to focus on lowering the computational cost, improving the accuracy of forecasts, and devising energy efficient AI systems for better scalability and usability in practice.

## Conclusion and future scope

In this study, an intelligent solar tracking system powered by artificial intelligence (COMLAT) was introduced. It was designed to overcome the barriers of conventional tracking methods. The results validated that COMLAT significantly enhances energy output, tracking accuracy, and climatic adaptability using a hybrid integration of CNN-LSTM for predicting the climate, XGBoost for energy output prediction, and Deep Q-Learning (DQL) for optimization of tracking in real time. Experimental testing proves that COMLAT is 55% more energy-efficient than fixed-tilt systems and 15–20% more energy-efficient than conventional dual-axis tracking. Compared to conventional fixed or pre-programmed movement systems, COMLAT dynamically changes its tracking angles based on real-time sensor data and AI forecasting to optimize the harvesting of solar energy under any environmental condition. The benchmarking results validate COMLAT’s superiority of COMLAT over cutting-edge tracking methods in terms of minimizing energy losses under cloudy weather, optimizing tracking decisions under mixed seasonal conditions, and supporting rapid real-time adaptability. Although AI-based tracking incurs computational overhead, COMLAT achieves sub-second decision latency, making it an efficient and scalable approach. This research also emphasizes the prospects of COMLAT in massive solar farms, smart grids, hybrid renewable systems, and space-based solar tracking, further supporting its industrial and commercial feasibility. In summary, the results confirmed that COMLAT is a revolutionary breakthrough in AI-based solar tracking, providing a smart, self-optimizing, and extremely efficient solution for new-generation renewable energy systems.

The future scope of the research, which will try to improve further in terms of computational complexity, includes energy storage systems and to enhance the self-learning capability of the proposed work. future work can investigate the hybridization of COMLAT with bio-inspired algorithms such as Firefly Optimization and Whale Optimization in order to improve adaptability within extreme climate zones. Moreover, incorporating federated learning to preserve the models privacy through model update contribution by the distributed solar farms along with practical real-world testing in multiple geographies can strengthen the system’s scalability, resiliency, and decision accuracy.

## Data Availability

The data required to reproduce the above findings are available within this research article.

## References

[1] Malkawi, A. M. A. et al. Maximum power point tracking enhancement for PV in microgrids systems using dual artificial neural networks to estimate solar irradiance and temperature. *Results Eng.* **25**, 104275. 10.1016/j.rineng.2025.104275 (2025).

[2] Nwokolo, S. C. et al. Machine learning and physics-based model hybridization to assess the impact of climate change on single- and dual-axis tracking solar-concentrated photovoltaic systems. *Phys. Chem. Earth Parts A/B/C*. **138**, 103881. 10.1016/j.pce.2025.103881 (2025).

[3] Abdul-Rahaim, L. A., Abdullah, A. A. & Aquraishi, A. K. L. Machine learning-based solar energy forecasting with climate adaptation. *Renew. Energy*. **215**, 1203–1215. 10.1016/j.renene.2023.07.014 (2023).

[4] Ahmad, M., Khan, R. & Shah, M. The impact of climate change on solar PV efficiency: A data-driven approach. *Energy Rep.* **9**, 233–245. 10.1016/j.egyr.2023.04.011 (2023).

[5] Ali, M. I. & Rashid, H. IoT-based smart solar PV monitoring and prediction using AI. *IEEE Trans. Sustain. Energy*. **14** (3), 1247–1258. 10.1109/TSTE.2023.3285612 (2023).

[6] Amer, Q. & Qouneh, R. Deep learning for solar energy forecasting under climate variability. *Appl. Energy*. **345**, 120932. 10.1016/j.apenergy.2023.120932 (2023).

[7] Augustine, C. & Lopez, A. Climate change effects on concentrated photovoltaic performance. *Sol. Energy*. **225**, 98–110. 10.1016/j.solener.2024.05.002 (2024).

[8] Chandra, R., Sharma, A. & Patel, K. AI-driven solar power optimization using tracking systems. *J. Renew. Sustain. Energy*. **15** (4), 4321–4333. 10.1063/5.0147893 (2023).

[9] Folgado, F. J., Garcia, M. & Herrero, R. IoT and machine learning for adaptive solar energy harvesting. *Energy AI*. **12**, 100164. 10.1016/j.egyai.2023.100164 (2023).

[10] González, H., Martinez, L. & Perez, R. Single-axis vs dual-axis solar tracking in climate-affected regions. *Sol. Energy*. **194**, 312–323. 10.1016/j.solener.2021.01.022 (2021).

[11] Jaafar, S. S., Yusof, A. M. & Osman, M. Performance evaluation of solar trackers in extreme weather conditions. *Renew. Sustain. Energy Rev.* **168**, 112953. 10.1016/j.rser.2024.112953 (2024).

[12] Jenie, Y. I. & Hartanto, W. Adaptive control of solar tracking systems using AI. *IEEE Access.* **11**, 23841–23852. 10.1109/ACCESS.2023.3247932 (2023).

[13] Krismanto, A. U. & Nugroho, D. Solar energy optimization via reinforcement learning. *J. Clean. Prod.* **390**, 136709. 10.1016/j.jclepro.2023.136709 (2023).

[14] Kumar, N., Gupta, A. & Singh, B. Machine learning models for solar power generation forecasting. *Energy Build.* **321**, 112435. 10.1016/j.enbuild.2023.112435 (2023).

[15] Louwen, A. & Schill, W. Long-term climate effects on solar PV and CPV systems. *Renew. Energy*. **312**, 127654. 10.1016/j.renene.2023.127654 (2023).

[16] Luque, A. & Hegedus, S. *Photovoltaic Science and Engineering* (Springer, 2023).

[17] Mohanty, S. P. & Khandelwal, A. AI and IoT for smart solar tracking and climate adaptation. *IEEE Trans. Smart Grid*. **15** (1), 112–125. 10.1109/TSG.2023.3245623 (2023).

[18] Nemet, G. F. & Trainor, A. Climate change effects on solar PV performance. *J. Solar Energy*. **215**, 98–115. 10.1016/j.solener.2023.07.009 (2023).

[19] Perez, R. & Hoff, T. AI-based solar energy prediction with climate variability considerations. *Sol. Energy*. **190**, 456–468. 10.1016/j.solener.2022.04.012 (2022).

[20] Rohit, A. & Mukherjee, S. Neural networks in solar power generation forecasting. *IEEE Trans. Neural Networks*. **35** (2), 654–667. 10.1109/TNNLS.2024.3217890 (2024).

[21] Suganthi, L. & Samuel, A. A. Machine learning algorithms in renewable energy forecasting. *Renew. Energy Reviews*. **125**, 106432. 10.1016/j.rser.2023.106432 (2023).

[22] Wang, Z. & Zhou, L. Deep learning models for solar PV output forecasting. *Energy AI*. **15**, 100289. 10.1016/j.egyai.2024.100289 (2024).

[23] Wild, M. & Folini, D. Solar radiation variability due to climate change. *Clim. Dyn.* **62** (3), 781–795. 10.1007/s00382-022-06153-1 (2022).

[24] Zhang, T. & Li, J. Effect of climate extremes on solar energy systems. *Renew. Energy*. **210**, 104–115. 10.1016/j.renene.2024.05.010 (2024).

[25] Jerez, S. & Trigo, R. The impact of climate change on solar resource availability. *Renew. Energy*. **214**, 451–463. 10.1016/j.renene.2023.06.032 (2023).

[26] Bukhsh, Q. & Malik, M. Performance analysis of single-axis vs dual-axis solar trackers. *J. Sol. Energy Eng.* **143** (5), 051001. 10.1115/1.4053712 (2023).

[27] Bagher, A. M. Comparative study of fixed, single, and dual-axis solar tracking systems. *Sol. Energy*. **235**, 120–134. 10.1016/j.solener.2023.01.015 (2023).

[28] Chakraborty, S. & Bhattacharya, S. AI-driven energy management in solar PV systems. *IEEE Access.* **11**, 13027–13038. 10.1109/ACCESS.2023.3246783 (2023).

[29] Patel, D. & Shah, V. Solar PV performance optimization using machine learning models. *Energy AI*. **11**, 100267. 10.1016/j.egyai.2023.100267 (2023).

[30] Verma, A. & Joshi, P. Sustainable energy solutions using AI for solar PV efficiency. *J. Renew. Energy*. **78**, 89–102. 10.1016/j.jre.2024.03.005 (2024).

[31] Wei, C. & Chen, Y. Impact of climate conditions on photovoltaic energy conversion. *J. Renew. Energy Res.* **15** (3), 198–210. 10.1016/j.jre.2023.05.007 (2023).

[32] Yang, D. & Lu, H. Optimizing solar energy production using AI-driven control mechanisms. *Renew. Energy*. **223**, 345–359. 10.1016/j.renene.2024.03.012 (2024).

[33] Zhao, F. & Liu, M. Performance evaluation of solar tracking systems under dynamic climate conditions. *Sol. Energy*. **217**, 101–115. 10.1016/j.solener.2023.06.009 (2023).

[34] Sayeef, S. & Blakers, A. Dual-axis solar tracking and climate resilience: A case study. *Appl. Energy*. **362**, 123104. 10.1016/j.apenergy.2023.123104 (2023).

[35] Khalil, M. & Abbas, K. A review on climate adaptation in solar PV systems using machine learning techniques. *Renew. Sustain. Energy Rev.* **154**, 112014. 10.1016/j.rser.2023.112014 (2023).

[36] Shen, W. & Huang, T. AI-based climate change impact prediction on solar photovoltaic efficiency. *IEEE Trans. Sustain. Energy*. **14** (2), 1127–1140. 10.1109/TSTE.2023.3245689 (2023).

[37] Hassan, M. & Rahman, M. Comparative study of AI-driven solar forecasting models under changing climate conditions. *J. Renew. Sustain. Energy*. **15** (3), 3392–3410. 10.1063/5.0123478 (2023).

[38] Ozturk, M. & Karadogan, E. Impact of extreme weather on solar tracking systems: A machine learning approach. *Renew. Energy*. **219**, 541–555. 10.1016/j.renene.2023.08.021 (2023).

[39] Espinosa, M. & Rubio, J. Smart energy grid management integrating AI and solar tracking systems. *Energy AI*. **14**, 100276. 10.1016/j.egyai.2023.100276 (2023).

[40] Liu, X. & Fang, Y. Deep learning-based solar radiation forecasting with climate factors. *J. Clean. Prod.* **398**, 136847. 10.1016/j.jclepro.2023.136847 (2023).

[41] Zafar, J. & Ahmad, T. Analysis of temperature variations on photovoltaic efficiency using AI models. *Sol. Energy*. **218**, 135–149. 10.1016/j.solener.2023.07.015 (2023).

[42] Navarro, E. & Jiménez, A. Climate-sensitive solar energy forecasting using machine learning techniques. *Energy Rep.* **9**, 567–580. 10.1016/j.egyr.2023.06.032 (2023).

